# Molecular Evolution of the HMPV-A *Fusion* Gene Reveals Lineage-Specific Diversification and Structural Conservation

**DOI:** 10.3390/microorganisms14102386

**Published:** 2026-10-18

**Authors:** Kazuhiko Harada, Fuminori Mizukoshi, Tatsuya Shirai, Ryusuke Kimura, Koo Nagasawa, Kazuto Ito, Isao Kurosawa, Kazuya Shirato, Akihide Ryo, Hirokazu Kimura

**Affiliations:** 1 Department of Health Science, Gunma Paz University Graduate School, Takasaki-shi 370-0006, Gunma, Japan; k_harada@bishinkai.or.jp; 2 Department of Clinical Medical Laboratory, Medical Corporation Bishinkai, Kurosawa Hospital, Takasaki-shi 370-1203, Gunma, Japan; 3 Department of Bioinformatics and Integrative Omics, National Institute of Infectious Diseases, Japan Institute for Health Security, Musashimurayama-shi 208-0011, Tokyo, Japan; mizukoshi.f@jihs.go.jp (F.M.); ryo.a@jihs.go.jp (A.R.); 4 Department of Respiratory Medicine, Faculty of Medicine, Kyorin University, Mitaka-shi 181-8611, Tokyo, Japan; 5 Department of Health Science, Gunma Prefectural Institute of Public Health and Environmental Sciences, Maebashi-shi 371-0052, Gunma, Japan; kimura-r56@pref.gunma.lg.jp; 6 Department of Pediatrics, Graduate School of Medical Science, Chiba University, Chiba-shi 260-8670, Chiba, Japan; k-nagasawa@chiba-u.jp; 7 Department of Urology, Medical Corporation Bishinkai, Kurosawa Hospital, Takasaki-shi 370-1203, Gunma, Japan; k_ito@bishinkai.or.jp (K.I.); i_kurosawa@bishinkai.or.jp (I.K.); 8 Advanced Medical Science Research Center, Gunma Paz University, Takasaki-shi 370-0006, Gunma, Japan; 9 Department of Respiratory Viruses, National Institute of Infectious Diseases, Japan Institute for Health Security, Musashimurayama-shi 208-0011, Tokyo, Japan; shirato.k@jihs.go.jp

**Keywords:** human metapneumovirus genotype A (HMPV-A), *fusion* (*F*) gene, molecular evolution, phylodynamics, structural conservation

## Abstract

Human metapneumovirus genotype A (HMPV-A) comprises genetically diverse lineages, but whether *F*-gene diversification is accompanied by changes in the conserved fusion (F) protein remains unclear. We analyzed 500 PARNAS-selected HMPV-A *F* gene sequences from 25 countries collected between 1982 and 2025 using time-scaled phylogenetics, Bayesian skyline analysis, evolutionary-rate and patristic-distance estimation, codon-based selection analysis, sequence-identity profiling, and structural epitope modeling. Three phylogenetically distinct clusters were identified. Cluster 1 diverged from the common ancestor of Clusters 2 and 3 around 1927. Cluster 3 comprised 435 representative sequences and showed the highest estimated evolutionary rate, greatest phylogenetic diversity, and the most pronounced recent demographic fluctuations. The overall evolutionary rate was 9.20 × 10^−4^ substitutions/site/year. Despite nucleotide-level diversification, whole-protein identity exceeded 97% in all clusters, 299 codons (approximately 55%) were consistently under negative selection, whereas positive-selection signals were limited and method-specific, with MEME identifying codons 188 and 271 and FUBAR identifying codon 528. Major neutralizing antibody-binding regions were largely conserved, and the six modeled representatives showed similar global F-protein backbone structures and predicted epitope-surface distributions, although A61S and K143T occurred within neutralizing antibody-binding sites and require experimental evaluation. These findings demonstrate lineage-specific *F* gene diversification under strong functional constraint and support the HMPV F protein as a broadly conserved intervention target.

## 1. Introduction

Human metapneumovirus (HMPV) is an enveloped, non-segmented, negative-sense RNA virus belonging to the family *Pneumoviridae*, genus *Metapneumovirus* [1]. Since its identification in 2001, HMPV has been recognized as an important cause of acute respiratory tract infection worldwide, particularly in infants and young children, older adults, and immunocompromised individuals [1,2,3]. Clinical disease ranges from mild upper respiratory tract illness to bronchiolitis and pneumonia requiring hospitalization [2,3]. Despite this substantial disease burden, no licensed HMPV vaccine or virus-specific antiviral therapy is currently available, and clinical management remains largely supportive [4]. Defining the long-term evolution of viral proteins that mediate entry and are targeted by protective antibodies is therefore relevant to molecular surveillance and to the development of preventive interventions.

HMPV is divided into two major genetic groups, HMPV-A and HMPV-B, each containing multiple genotypes and lineages [5,6,7]. HMPV-A has undergone substantial lineage diversification and turnover, particularly within the A2 group [8,9]. Historically, HMPV-A has been classified into A1 and A2, with A2 further subdivided into A2a, A2b, and A2c, whereas more recent nomenclature systems use numerical lineage designations, including A2.1- and A2.2-derived types [5,7,9]. A2c-related viruses carrying nucleotide duplications in the attachment glycoprotein *G* gene have disseminated internationally and illustrate the continuing genomic diversification of HMPV-A [10,11,12]. These changes in the highly variable *G* gene raise the question of whether contemporaneous diversification is also detectable in the more functionally constrained *F* gene.

The fusion glycoprotein (F) is essential for HMPV entry because it mediates fusion of the viral envelope with the host-cell membrane and is a principal target of neutralizing antibodies [4,13]. In contrast to the highly variable G protein, the F protein is comparatively conserved, which has made it a major antigenic target for vaccine design and antibody-based prevention [4,14,15]. The pneumovirus F protein contains several defined antigenic regions, including sites Ø and I–V [16,17,18]. Some of these regions are structurally conserved across pneumoviruses, whereas local amino acid variation, glycosylation, and conformational context can modulate antibody recognition [16,17,18]. Consequently, even infrequent substitutions in a globally conserved F protein may be relevant if they occur within or near neutralizing antibody-binding sites or structurally exposed regions.

The evolutionary behavior of the HMPV *F* gene may differ substantially from that of the *G* gene [12,13,14]. Although *G*-gene variation has been used extensively for molecular epidemiological classification, the *F* gene is more directly linked to membrane fusion and neutralizing immunity [4,13,14,15]. It therefore remains important to determine whether phylogenetically distinct HMPV-A *F*-gene lineages differ in substitution rate, genetic diversity, or demographic history and, critically, whether such nucleotide-level diversification is accompanied by persistent amino acid changes, altered selective pressures, or detectable changes in predicted antigenic regions. Resolving this distinction is necessary to separate lineage diversification from antigenically meaningful evolution of the F protein.

Our previous analysis of the HMPV-B *F* gene provides a comparative framework for this question [19]. HMPV-B1 and HMPV-B2 showed distinct evolutionary and demographic histories, whereas F-protein sequence variation remained limited, purifying selection predominated, and substitutions within neutralizing antibody-binding regions were not predicted to produce major changes in conformational epitope architecture [19]. However, those findings cannot be assumed to apply to HMPV-A, which has a different lineage structure and more complex A2-derived diversification [9,10,11,12]. A corresponding lineage-focused analysis of HMPV-A is therefore needed to determine whether strong F-protein constraint is shared across the major HMPV genetic groups.

In the present study, we characterized the molecular evolution of the HMPV-A *F* gene using an integrated phylogenetic, phylodynamic, selection, sequence-conservation, and structural approach. Time-scaled Bayesian phylogenetics and minimum-spanning network analysis were used to characterize lineage relationships, whereas Bayesian skyline analysis, evolutionary-rate estimation, and patristic distances were used to assess lineage-specific demographic and genetic patterns. Codon-based analyses evaluated positive and negative selection, and sequence-identity profiling, structural modeling, and conformational B-cell epitope mapping were used to assess conservation of the F protein and established neutralizing antibody-binding regions. We specifically sought to determine whether lineage-specific diversification of the HMPV-A *F* gene is accompanied by evidence of adaptive amino acid evolution or detectable disruption of the conserved structural and predicted antigenic features of the F protein.

## 2. Materials and Methods

### 2.1. Sequence Data Collection and Dataset Construction

Human metapneumovirus (hMPV) nucleotide sequences were retrieved from the NCBI Virus database on 29 July 2026 using Metapneumovirus hominis (taxid: 3048148). Sequences with available collection dates and nucleotide lengths of ≥1600 nt were selected, yielding 2859 records. Records annotated as “UNVERIFIED” or “MAG” and sequences derived from non-human hosts were excluded. The reference sequence NC_039199.1 was additionally included, and its year of detection (2000) and country (The Netherlands) were assigned based on the original report [1].

The retrieved sequences were aligned with the reference *F* gene sequence using MAFFT v7.526 [20], and the region corresponding to the complete *F* gene was extracted. Sequences lacking substantial portions of the *F* gene, containing ambiguous nucleotides within the F protein-coding region, or showing abnormal translation were excluded. Sequences lacking only the terminal stop codon, or containing ambiguous nucleotides restricted to the stop codon, were retained when the complete F protein-coding sequence could be unambiguously translated. After quality control, 2399 complete *F* gene sequences remained.

Genotypes and clades were assigned using Nextclade v3.21.2 [21] with the hMPV dataset based on NC_039199.1 (https://clades.nextstrain.org/?dataset-name=nextstrain/hmpv/all-clades/NC_039199; accessed on 30 July 2026). Of these, 1222 sequences were classified as genotype A and were retained for further analysis.

The genotype A sequences were additionally examined using nucleotide- and amino-acid-based maximum-likelihood phylogenies constructed with IQ-TREE v3.0.1 [22]. Sequences showing unusual localized deletion and/or substitution patterns within the *F* gene, as well as those showing discordant and unstable phylogenetic placement between the A2.2.1 and A2.2.2 lineages, were excluded during sequence quality assessment.

Completely identical nucleotide sequences were identified by exact comparison of their complete *F* gene sequences using Python v3.11.5. This procedure resulted in 930 non-redundant sequences. These sequences were further subsampled using PARNAS v0.1.7 [23], with NC_039199.1 retained as a prior representative. A target dataset of 500 phylogenetically representative sequences was selected for the downstream analyses, retaining 97.17% of the overall phylogenetic diversity of the 930-sequence dataset.

Details of the sequences excluded during PARNAS-based down-selection are provided in Supplementary Table S1. The final dataset comprised 500 sequences collected from 25 countries between 1982 and 2025. Accession numbers, countries and years of detection, Nextclade clade assignments, and cluster assignments defined in this study for the 500 sequences are provided in Supplementary Figure S1 and Supplementary Table S2. These 500 sequences constituted the final dataset used in all subsequent analyses. The selected sequences were subsequently classified into three phylogenetically defined *F*-gene clusters, comprising 21 sequences in Cluster 1, 44 sequences in Cluster 2, and 435 sequences in Cluster 3.

### 2.2. Time-Scaled Phylogenetic Tree

A time-scaled phylogenetic tree of the HMPV-A *F* gene was constructed using the Bayesian Markov chain Monte Carlo (BMCMC) approach implemented in BEAST version 2.7.7 [24]. The analysis included the final dataset of 500 HMPV-A sequences. Before the BMCMC analysis, the optimal nucleotide substitution model (GTR+I+G) was determined using jModelTest2 version 2.1.10 (v20160303) [25]. Candidate combinations comprising six molecular clock models (strict clock, relaxed clock exponential, relaxed clock lognormal, random local clock, optimized relaxed clock, and fast relaxed clock lognormal) and two coalescent tree priors (constant population and exponential population) were compared using path-sampling/stepping-stone model comparison implemented in BEAST. The corresponding log marginal likelihood estimates are provided in Supplementary Table S11. The random local clock combined with the constant-population coalescent prior had the highest log marginal likelihood and was therefore selected for the final analysis.

The BMCMC chain was run for 100 million states, with parameters and trees sampled every 1000 states. Convergence and mixing were assessed by visual inspection of the trace plots and calculation of effective sample sizes (ESSs) using Tracer version 1.7.2 [26]. Based on the trace plots, the first 30% of samples were discarded as burn-in. After burn-in, ESS values exceeded 200 for all global parameters relevant to the reported overall tree estimates, including the posterior, likelihood, prior, tree height, tree length, mean clock rate, and coalescent population-size parameters. Four branch-associated clock-rate parameters, 15 individual random-local-clock indicator parameters, and the total number of rate shifts represented by sum(Indicators.) had ESS values below 200 (Supplementary Table S4). These low-ESS parameters were associated with branch-specific features of the random local clock. Accordingly, branch-specific substitution rates and the number, location, or magnitude of individual rate shifts were not reported or interpreted. Although the global posterior quantities used for the reported overall tree estimates had ESS values >200, these satisfactory ESS values were not taken to imply complete convergence of all local-clock parameters.

The post-burn-in posterior tree sample was summarized using TreeAnnotator version 2.7.6 to generate a maximum clade credibility (MCC) tree, with node heights set to Common Ancestor heights. The resulting tree was visualized using FigTree version 1.4.4 (http://tree.bio.ed.ac.uk/software/figtree/ accessed on 10 June 2026), and uncertainty in node-age estimates was expressed using 95% highest posterior density (HPD) intervals. Posterior probability values were displayed at internal nodes on an additional annotated version of the MCC tree using FigTree version 1.4.4. Branches were colored according to node posterior probability using an automatically scaled gradient. For downstream analyses, three major monophyletic clades identified in the MCC tree were operationally designated Clusters 1, 2, and 3. Their correspondence with the available Nextclade assignments was examined using the clade annotations obtained for each sequence. These cluster labels were used as an analytical framework and were not intended to constitute a new HMPV nomenclature.

### 2.3. Minimum-Spanning Network Construction

Minimum-spanning network analysis was performed to visualize mutational relationships among the 500 PARNAS-selected HMPV-A *F*-gene sequences [27]. The aligned *F*-gene sequences were imported into DnaSP version 6.12.03 [28], and a NEXUS haplotype data file was generated for network analysis. All 500 sequences were included in the analysis, and alignment sites containing gaps were excluded. Because sequences with 100% nucleotide identity had been removed during data curation, each haplotype represented a single unique *F*-gene sequence. The NEXUS file was imported into PopART version 1.7 [29], and a minimum-spanning network was constructed using the default minimum-spanning algorithm. Edges represented the minimum nucleotide differences required to connect sampled haplotypes. Geographic origin was assigned as a trait and displayed by node color. The network was used as a descriptive representation of sequence variation and genetic relationships within the selected dataset and was not interpreted as evidence of direct transmission, ancestor–descendant relationships, or lineage prevalence.

### 2.4. Bayesian Skyline Analysis and Substitution-Rate Estimation

Historical population dynamics were inferred separately for the three phylogenetically defined HMPV-A clusters (Clusters 1, 2, and 3) from *F*-gene sequences using the coalescent Bayesian skyline model implemented in BEAST version 2.7.7 [24]. Cluster-specific analyses were performed rather than combining the phylogenetically distinct clusters into a single demographic reconstruction.

The optimal nucleotide substitution model for each cluster was determined using jModelTest2 version 2.1.10 (v20160303) [25], and molecular clock models were selected using model-comparison procedures implemented in BEAST. The final model combinations were TrN+G with a strict molecular clock for Cluster 1, TPM2uf+I with an uncorrelated lognormal relaxed clock for Cluster 2, and HKY+I+G with a strict molecular clock for Cluster 3. The coalescent Bayesian skyline prior was used for all three clusters.

For Clusters 1 and 2, BMCMC analyses were conducted using a chain length of 300 million states. For Cluster 3, two independent BMCMC runs of 300 million states each were conducted, and the post-burn-in samples were combined. Parameters and trees were sampled every 1000 states, and the first 10% of each run was discarded as burn-in. Convergence and mixing were assessed using Tracer version 1.7.2 [26]. ESS values exceeded 200 for all nonconstant parameters used for the reported Bayesian skyline and substitution-rate estimates, including all population-size and group-size parameters (Supplementary Table S4). Bayesian skyline plots were reconstructed and visualized using Tracer, with uncertainty expressed using 95% HPD intervals.

Molecular evolutionary rates (substitutions/site/year) were obtained from the BEAST log files according to the molecular clock model applied. The posterior estimates and their 95% HPD intervals were reported for each cluster. No frequentist statistical tests were performed on the MCMC posterior samples. Detailed parameter settings and convergence statistics for the BSP analyses are provided in Supplementary Tables S3 and S4, respectively.

To assess the sensitivity of the Cluster 3 phylodynamic inference to uneven temporal sampling, a temporally balanced Cluster 3 dataset was additionally constructed. For each collection year containing more than 10 sequences, PARNAS v0.1.7 [23] was used to retain up to 10 phylogenetically representative sequences, whereas all sequences were retained for years containing 10 or fewer sequences. This procedure reduced Cluster 3 from 435 to 177 sequences. The temporally balanced dataset was analyzed using the same BEAST-based framework as the original Cluster 3 dataset. Molecular-clock model selection was performed by path-sampling/stepping-stone analysis, and the best-supported model was used for the final Bayesian skyline and substitution-rate analyses. Convergence and mixing were assessed using the same criteria as for the primary analyses. Detailed model settings, convergence statistics, and marginal likelihood estimates are provided in Supplementary Tables S3, S4, and S11, respectively.

### 2.5. Phylogenetic Distance Analysis

We assessed the genetic diversity of the HMPV-A *F* gene by calculating patristic distances from phylogenetic trees. Maximum likelihood (ML) phylogenetic trees were independently reconstructed for four datasets: (i) all 500 HMPV-A strains, (ii) Cluster 1, (iii) Cluster 2, and (iv) Cluster 3. The ML trees were constructed using MEGA version 7.0 [30], which was also used to identify the optimal nucleotide substitution model for each dataset. The best-fitting nucleotide substitution model was selected according to the lowest Bayesian Information Criterion value. GTR+I+G was selected for the complete dataset, whereas TN93+G was selected for Clusters 1 and 2, and TN93+I+G for Cluster 3. The nearest-neighbor interchange (NNI) algorithm was used for tree searching, and gaps or missing data were handled using complete deletion. Nodal support was evaluated using 1000 bootstrap replicates. All pairwise patristic distances, representing the number of nucleotide substitutions per site, were then calculated from each tree using PATRISTIC version 1.0 [31]. The resulting distance distributions were visualized as violin plots using Orange Data Mining version 3.35.0 [32]. Mean, median, and interquartile range (IQR) values were reported as descriptive statistics. Statistical tests comparing all pairwise distances were not performed because the distances are not independent observations.

### 2.6. Analysis of Codon-Specific Selective Pressures

We evaluated codon-specific selective pressures acting on the HMPV-A F protein by analyzing all 500 *F*-gene coding sequences (1617 bp, corresponding to 539 amino acids). Nonsynonymous (dN) and synonymous (dS) substitution rates at each codon were estimated using four methods implemented on the Datamonkey web server (https://www.datamonkey.org; accessed on 29 March 2026, 19 September 2026) [33]: Single-Likelihood Ancestor Counting (SLAC) [34], Fixed Effects Likelihood (FEL) [34], Fast Unconstrained Bayesian Approximation (FUBAR) [35], and the Mixed Effects Model of Evolution (MEME) [36]. For SLAC, FEL, and MEME, statistical significance was defined as *p* < 0.05; for FUBAR, a posterior probability > 0.9 was used as the significance threshold. Codons detected by an individual method were treated as method-specific candidate positively selected sites, whereas codons supported by all four methods were designated high-confidence positively selected sites. Codons supported as being under negative selection by SLAC, FEL, and FUBAR were considered high-confidence negatively selected sites. MEME was not used to identify negatively selected sites because it is designed to test for episodic or pervasive diversifying selection. To assess whether the candidate positive-selection signals detected in the complete dataset were disproportionately influenced by the numerical predominance of Cluster 3, positive-selection analyses were additionally performed separately for Clusters 1, 2, and 3 using the same four methods and significance criteria. The same analyses were also applied to the temporally balanced Cluster 3 dataset used in the sampling-sensitivity analysis. Because the numbers of sequences differed substantially among clusters, particularly for Clusters 1 and 2, failure to detect a significant site in an individual cluster was not interpreted as evidence for the absence of diversifying selection.

### 2.7. Amino Acid Identity Analysis

We assessed HMPV-A F-protein sequence identity using SimPlot++ version 1.3, which calculates sequence identity using a sliding-window approach [37]. The translated sequence of the HMPV reference strain (GenBank accession: NC_039199.1) was designated as the query sequence, and the amino acid sequences assigned to Clusters 1, 2, and 3 were used as comparison sequences. For Cluster 1, the reference sequence itself was excluded from the calculation, leaving 20 comparison sequences. The analysis used a window size of 40 amino acids and a step size of 5 amino acids, with percentage identity as the similarity metric. Amino acid identity relative to the reference strain was also evaluated for the three clusters across (i) the full-length F-protein sequence and (ii) the core regions of neutralizing antibody-binding sites. For each cluster and analyzed region, amino acid identity was calculated as the percentage of residue comparisons that matched the reference sequence NC_039199.1 across all sequences and amino acid positions included in the analysis. Specifically, the total number of identical residues matches divided by the product of the number of sequences in the cluster and the total number of amino acid positions analyzed, multiplied by 100. The core regions of the neutralizing antibody-binding sites were defined from previous reports [16,17,18,19,38] as follows: site Ø (amino acids 54–65 and 170–183), site I (amino acids 23–31 and 282–284), site II (amino acids 233–238), site III (amino acids 277–281), site IV (amino acids 395–405), and site V (amino acids 136–156). Sequence divergence across the full-length F protein and each core region was calculated for each cluster, and similarity to the reference strain was compared among clusters. Furthermore, to assess amino acid conservation within the core regions of neutralizing antibody-binding sites, the frequency of the predominant amino acid was calculated independently for each of the three clusters. The frequency of the predominant amino acid at each position was defined as the percentage of sequences within a given cluster that contained the most frequent amino acid at that corresponding position. Specifically, this was calculated by dividing the number of sequences harboring the predominant amino acid by the total number of sequences analyzed in that cluster, and then multiplying by 100. Alternative amino acids and the numbers of sequences containing them are shown separately.

To further assess amino acid diversity independently of the single reference strain, consensus-based and pairwise amino acid identity analyses were performed using the full-length F-protein sequences. Analyses were conducted for the complete dataset and separately for Clusters 1, 2, and 3. For each dataset, amino acid sequences were aligned using the MAFFT online service, and the consensus amino acid sequence displayed in the MAFFT MSA Viewer was exported in FASTA format. Amino acid identity between each sequence and its corresponding consensus sequence was calculated in MEGA version 7.0 [30] using uncorrected p-distances and expressed as (1—p-distance) × 100. All pairwise amino acid identities among sequences within each dataset were calculated in the same manner. Consensus-based identity was summarized using the mean, median, and range, whereas pairwise identity was summarized using the mean, median, interquartile range, and range. These descriptive statistics were calculated using Microsoft Excel (Microsoft Office Home and Business 2024; Microsoft Corporation, Redmond, WA, USA). Pairwise comparisons for the complete dataset included both within- and between-cluster sequence pairs.

### 2.8. Structural Modeling

Representative strains were selected from each cluster for three-dimensional (3D) modeling of the HMPV-A F protein: Cluster 1, EU857542.1 (1982) and OL794389.1 (2006); Cluster 2, EU857564.1 (1986) and OL794442.1 (2016); and Cluster 3, JQ413393.1 (2002) and PV218011.1 (2024). Two representative sequences were selected from each cluster based on their collection years and phylogenetic positions in the amino-acid tree, so that temporally separated and phylogenetically distinct sequences were represented within each cluster. After selection, amino acid residues within the core regions of established neutralizing antibody-binding sites were compared with the cluster-specific consensus sequences shown in Supplementary Figure S3. For Cluster 1, EU857542.1 and OL794389.1 were selected as the earlier and later representatives, respectively, based on their collection years and phylogenetic positions. Subsequent residue comparison showed that EU857542.1 matched the cluster consensus at these sites, whereas OL794389.1 carried A61S. For Cluster 2, S61 and T143 were the predominant residues, whereas A61 and K143 were present in 27.27% and 31.82% of sequences, respectively. EU857564.1 and OL794442.1 were selected as the earlier and later representatives, respectively, based on their collection years and phylogenetic positions. Subsequent residue comparison showed that EU857564.1 carried the alternative A61/K143 combination together with N404S, whereas OL794442.1 matched the cluster consensus at these sites. For Cluster 3, JQ413393.1 and PV218011.1 were selected as the earlier and later representatives, respectively, based on their collection years and phylogenetic positions. Subsequent residue comparison showed that JQ413393.1 matched the cluster consensus at these sites, whereas PV218011.1 carried the K171R substitution.

Structural models of the HMPV-A F protein were generated using LocalColabFold version 1.5.3 in a local computing environment [39]. Before structure prediction, amino acids 1–18 at the N-terminus were removed from each F-protein sequence based on the experimentally determined HMPV F structures available in the Protein Data Bank (PDB IDs: 8W3Q and 5WB0). Structure prediction was performed in two stages. First, multiple-sequence alignments in a3m format and template-search results were generated using colabfold_search. UniRef30 (release 2302), PDB100 (release 230517), and colabfold_envdb (release 202108) were used as the sequence, structural-template, and environmental-sequence databases, respectively. Template and environmental-sequence searches were conducted using MMseqs2 version 18.8cc5c with four CPU threads. Second, the resulting a3m files and PDB100 template-hit files were supplied to colabfold_batch for structure prediction. The principal options were --amber, --templates, --num-recycle 30, --use-gpu-relax, --pdb-hit-file, and --local-pdb-path.

Five candidate models were generated for each sequence. A representative model was selected by considering the overall prediction confidence and structural consistency, including the predicted local distance difference test (pLDDT), predicted template modeling score (pTM), and root mean square deviation (RMSD). For structural visualization, the C-terminal region beginning at residue 500 was omitted because it was predicted as an extended, poorly structured segment; consequently, residues 19–499 of the full-length F-protein sequence were displayed.

The selected structures were visualized using PyMOL version 3.0.3 [40] (https://www.pymol.org/) to examine the overall trimeric architecture and spatial distribution of antigenic sites. To quantify structural similarity among the representative strains, pairwise Cα RMSD values were calculated for all six models (two per cluster). RMSD calculations were performed in PyMOL version 3.0.3 using the align command, with the analysis restricted to Cα atoms.

### 2.9. B-Cell Epitope Prediction and Comparative Analysis

Conformational B-cell epitopes on the F-protein models of representative strains were predicted using four methods: DiscoTope 3.0 [https://services.healthtech.dtu.dk/services/DiscoTope-3.0/ (accessed on 10 June 2026)] (higher-confidence threshold: 1.50; recall up to approximately 30%) [41], ElliPro [https://tools.iedb.org/ellipro/ (accessed on 10 June 2026)] (cutoff: 0.5) [42], SEPPA 3.0 [http://www.badd-cao.net/seppa3/ (accessed on 10 June 2026)] (cutoff: 0.089) [43], and SEMA-3D implemented in SEMA 2.0 [https://github.com/AIRI-Institute/SEMAi/ (accessed on 10 June 2026)] (cutoff: 0.76) [44]. SEMA-3D predictions were performed using the publicly available Python implementation and the five released SaProt-based model weights, with each chain of the F-protein model analyzed independently and the prediction scores averaged across the five models. The web-based tools were accessed on 10 June 2026. An amino acid residue was considered a putative B-cell epitope when it was identified by at least three of the four methods. The predicted epitopes were subsequently mapped onto the three-dimensional protein structures using PyMOL. To further characterize epitope-associated variation, amino acid substitutions associated with predicted epitopes and experimentally determined neutralizing antibody-binding sites were visualized in a linear sequence alignment. The oldest strain in the dataset (a Cluster 1 strain collected in 1982; GenBank accession: EU857542.1) was used as the reference sequence for the linear representation.

## 3. Results

### 3.1. Time-Scaled Phylogeny and Divergence Time Estimation

**Figure 1:**
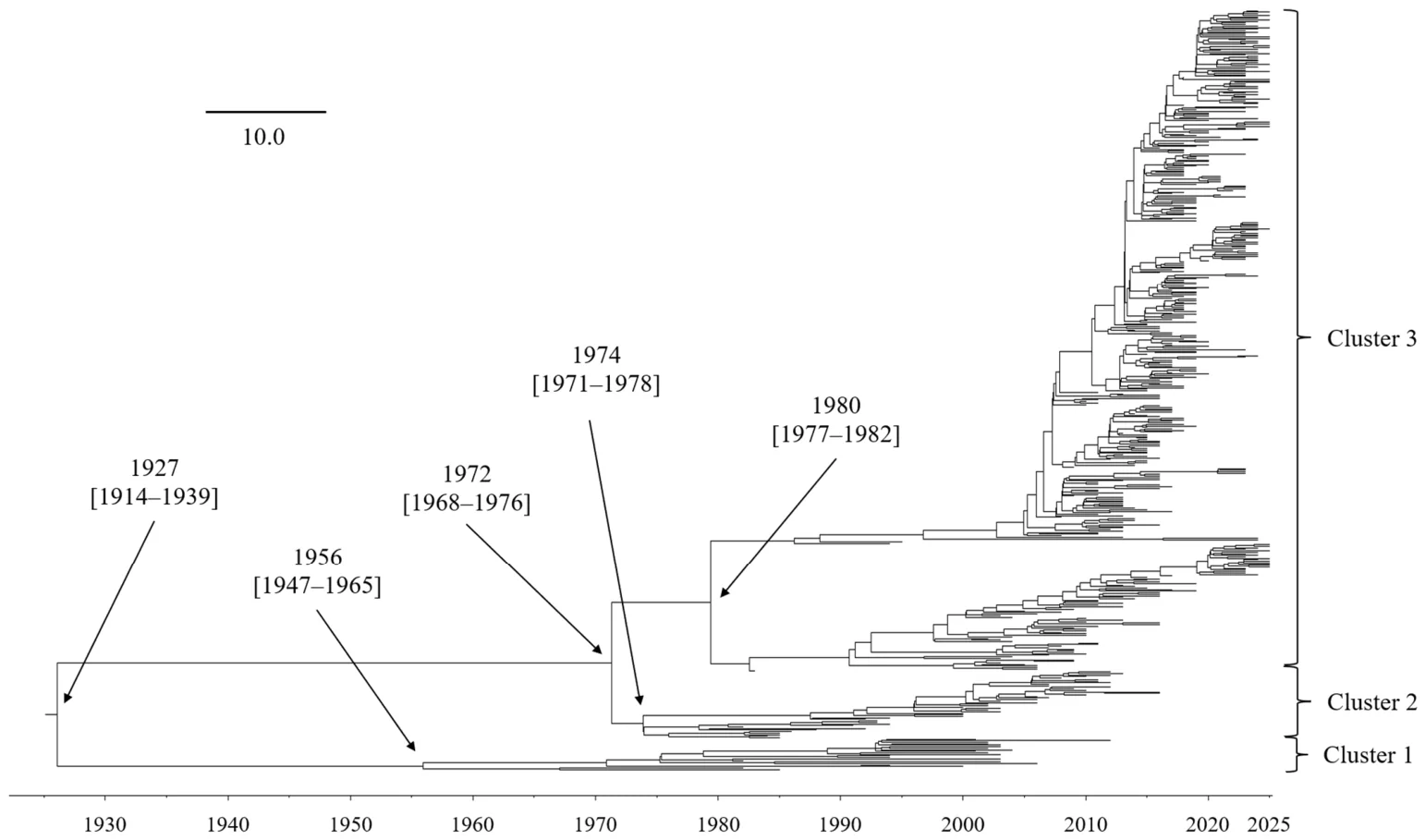
Time-scaled phylogenetic tree of the HMPV-A *F* gene inferred using the BMCMC method. The scale bar indicates time (in years). Numbers in parentheses denote the 95% HPD interval.

The time-scaled phylogenetic tree constructed using the BMCMC method revealed the evolutionary history of the HMPV-A *F* gene (Figure 1). Among the candidate molecular-clock and tree-prior combinations, the random local clock combined with the constant-population coalescent prior had the highest log marginal likelihood (−22,491.916) and was therefore selected for the final time-scaled phylogenetic analysis (Supplementary Table S11). Temporal sampling differed markedly among the three clusters. Cluster 1 comprised 21 sequences collected from 1982 to 2012, with a median collection year of 2002, whereas Cluster 2 comprised 44 sequences collected from 1982 to 2016, with a median year of 2003. Cluster 3 comprised 435 sequences collected from 1983 to 2025 and was strongly weighted toward recent years, with a median collection year of 2018. Only 6/21 (28.6%), 14/44 (31.8%), and 3/435 (0.7%) sequences in Clusters 1, 2, and 3, respectively, were collected before 2000. Detailed temporal and geographic compositions are provided in Supplementary Table S5. Cluster 1 is estimated to have diverged from the common ancestor of Clusters 2 and 3 around 1927 (95% highest posterior density [HPD]: 1914–1939). Subsequently, Cluster 1 began to diversify into multiple subclusters around 1956 (95% HPD: 1947–1965). The common ancestor of Clusters 2 and 3 is estimated to have begun diverging around 1972 (95% HPD: 1968–1976). Subsequently, Cluster 2 began to differentiate into multiple subclusters around 1974 (95% HPD: 1971–1978), and Cluster 3 began to differentiate into multiple subclusters around 1980 (95% HPD: 1977–1982). In particular, Cluster 3 underwent increased diversification and branched into multiple descendant branches. The major nodes defining Clusters 1–3 were strongly supported, with posterior probabilities of 1.000 (Supplementary Figure S5).

Cross-tabulation with the available Nextclade assignments showed a clear correspondence between the phylogenetically defined clusters and established HMPV-A lineages. All 21 Cluster 1 sequences were assigned to A1, and all 44 Cluster 2 sequences were assigned to A2.1. Cluster 3 comprised 4 A2.2, 84 A2.2.1, and 347 A2.2.2 sequences. Thus, Cluster 1 corresponds to A1 and Cluster 2 to A2.1, whereas Cluster 3 encompasses the A2.2-derived lineages A2.2, A2.2.1, and A2.2.2 (Supplementary Table S2). The cluster terminology was used as an operational framework for subsequent comparative analyses and was not intended to propose a new HMPV nomenclature.

After exclusion of the initial 30% of samples as burn-in, ESS values exceeded 200 for all global parameters relevant to the reported tree estimates. In contrast, four branch-associated clock-rate parameters, 15 individual random-local-clock indicator parameters, and sum(Indicators.) had ESS values below 200 (Supplementary Table S4). Therefore, branch-specific substitution rates and the number or locations of local rate shifts were not interpreted.

### 3.2. Minimum-Spanning Network Analysis

**Figure 2:**
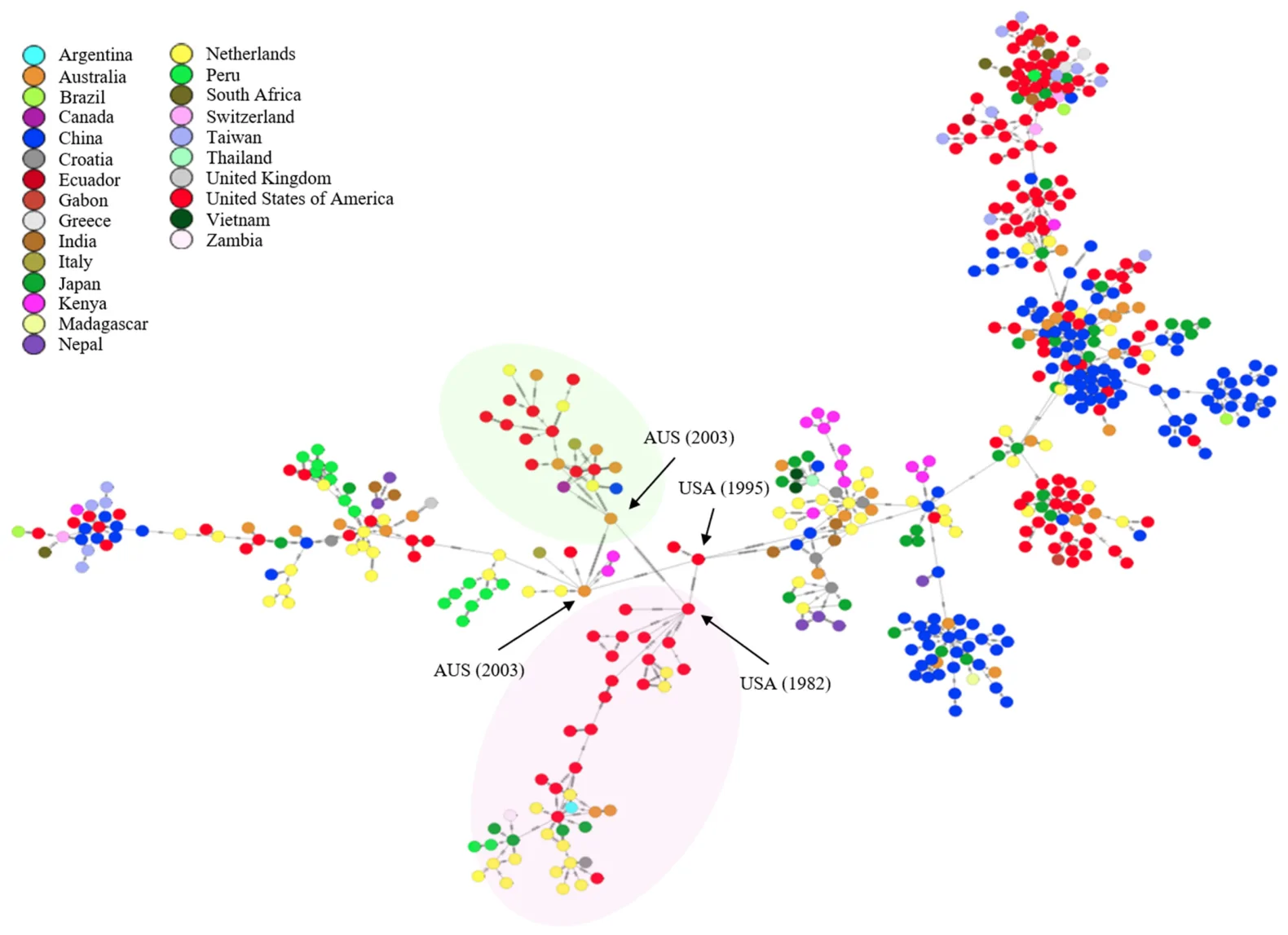
Minimum-spanning network of HMPV-A *F*-gene sequences collected from 25 countries. Each node represents a sequence and is colored according to the country of collection. Cluster assignments were defined according to the time-scaled phylogeny. Sequences assigned to Clusters 1 and 2 formed relatively compact regions of the network and are highlighted by light-green and light-purple ellipses, respectively, whereas Cluster 3 sequences were more broadly distributed and are therefore not enclosed by a single boundary. Arrows indicate the minimum-distance network connections between sequences assigned to different clusters. These connections reflect the structure of the minimum-spanning network and do not imply direct transmission or ancestor–descendant relationships.

Minimum-spanning network analysis clarified the distribution of sequence variation and genetic structure among HMPV-A strains (Figure 2). The network showed three groupings broadly consistent with the cluster assignments derived from the time-scaled phylogeny. Each node represented a single viral strain, and each connecting line indicated the number of mutational steps between strains. Cluster 1 comprised strains detected in six countries: Australia, Canada, China, Italy, the Netherlands, and the United States of America. Cluster 2 comprised strains detected in eight countries: Argentina, Australia, Croatia, Japan, the Netherlands, Peru, the United States of America, and Zambia. Cluster 3 comprised strains detected in 22 countries: Australia, Brazil, China, Croatia, Ecuador, Gabon, Greece, India, Italy, Japan, Kenya, Madagascar, Nepal, the Netherlands, Peru, South Africa, Switzerland, Taiwan, Thailand, the United Kingdom, the United States of America, and Vietnam. Clusters 1 and 2 formed relatively compact groupings, whereas Cluster 3 was distributed more diffusely across multiple branches. Inter-cluster connections were observed between Australian and USA strains for Clusters 1 and 2, between two USA strains for Clusters 2 and 3, and between Australian strains for Clusters 1 and 3. The minimum-distance edges connecting the clusters represented more than 10 nucleotide substitutions in the *F* gene. These connections were selected by the network algorithm and do not imply direct transmission or ancestral relationships among the strains. These findings demonstrate the coexistence of multiple *F*-gene lineages within the PARNAS-selected HMPV-A sequence dataset. The countries represented in each cluster and the corresponding numbers of strains are shown in Supplementary Table S5.

### 3.3. Historical Changes in Effective Population Size

**Figure 3:**
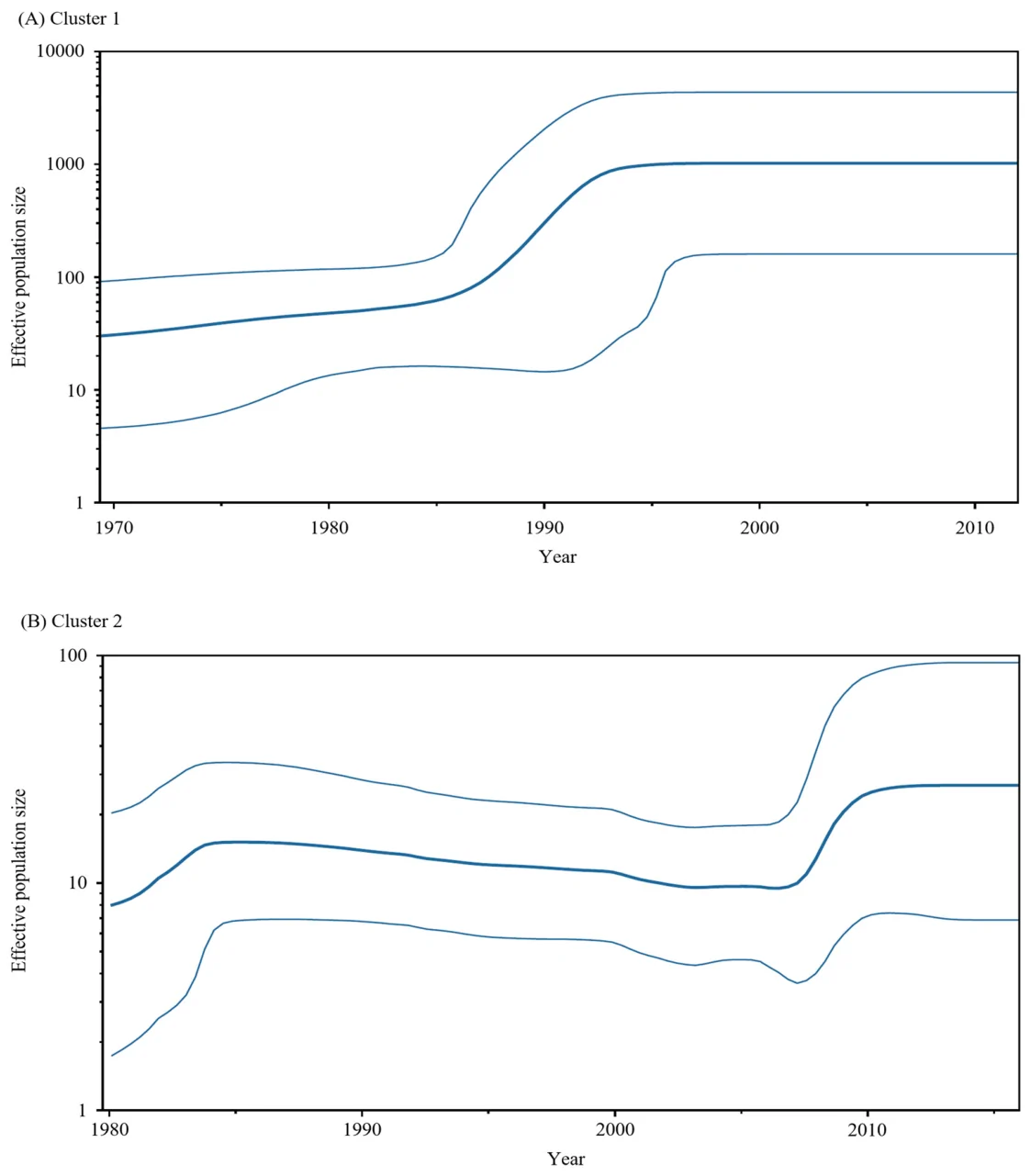

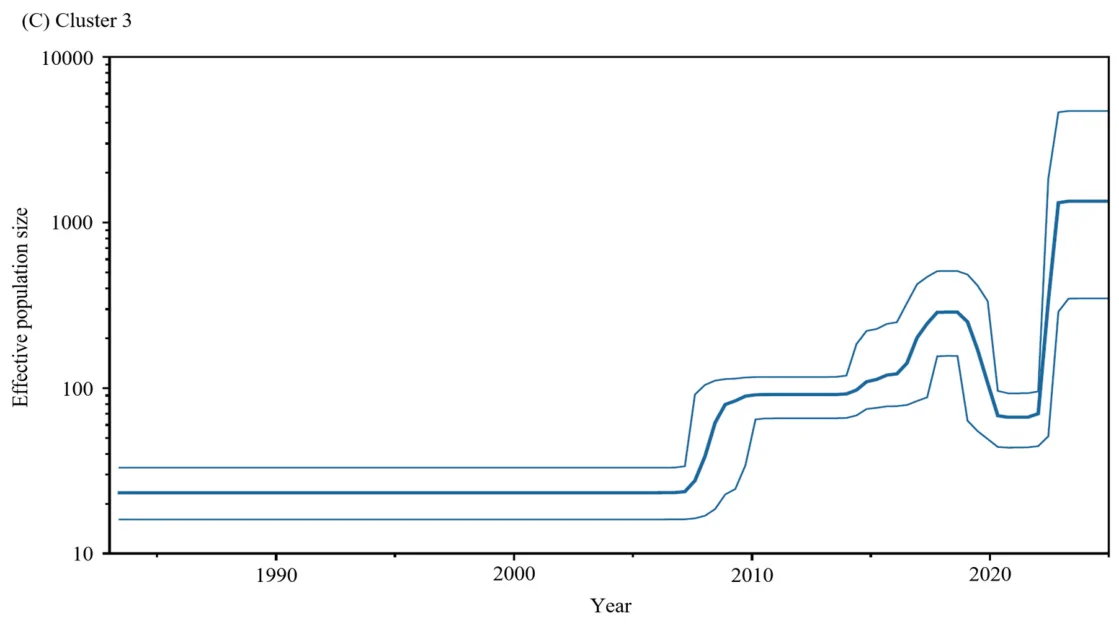
Bayesian skyline plot analyses of the HMPV-A *F* gene. The plots show the phylodynamic histories of (**A**) Cluster 1, (**B**) Cluster 2, and (**C**) Cluster 3. The *x*-axis represents calendar year, and the *y*-axis represents effective population size. The solid blue line indicates the posterior mean, and the thin blue lines represent the 95% highest posterior density (HPD) interval.

A BSP analysis of the HMPV-A *F* gene revealed temporal fluctuations in the virus’s effective population size (Figure 3). The three clusters exhibited distinct demographic trajectories. In Cluster 1, the effective population size increased modestly between 1970 and 1993, after which it remained relatively stable with little fluctuation (Figure 3A). In Cluster 2, the effective population size gradually increased from 1980 to 1984, followed by a gradual decline that continued until around 2006. It then increased gradually from 2007 to approximately 2010, slightly exceeding the levels observed previously. From 2010 onward, the effective population size remained relatively stable, with no major fluctuations (Figure 3B). In Cluster 3, the effective population size increased rapidly around 2007 and remained relatively stable until approximately 2015. A second sharp increase occurred in 2017, followed by a pronounced decline in 2019. The effective population size increased again around 2022, reaching levels that exceeded the previous peaks (Figure 3C).

To assess the potential effect of temporal sampling intensity on the Cluster 3 reconstruction, we performed a sensitivity analysis using a temporally balanced Cluster 3 dataset (tbCl3; *n* = 177), generated by retaining a maximum of 10 phylogenetically representative sequences per collection year using PARNAS. Model comparison again supported a strict molecular clock, and the same HKY+I+G substitution model class was selected. The tbCl3 Bayesian skyline retained the broad increase in inferred effective population size from the late 2000s onward and a recent increase near the end of the sampling period. However, the amplitude of the fluctuations was reduced, and the pronounced rise and decline observed in the late 2010s in the original Cluster 3 analysis were less evident after temporal balancing (Supplementary Figure S4). These findings indicate that the broad long-term demographic trend was retained, whereas some shorter-term skyline features were sensitive to temporal sampling intensity.

### 3.4. Estimated Evolutionary Rate

The evolutionary rate of the *F* gene across all HMPV-A strains was estimated using a time-scaled phylogenetic analysis implemented in BEAST. For each cluster, the evolutionary rate was estimated separately using Bayesian Skyline Plot (BSP) analysis.

The estimated evolutionary rate for all HMPV-A strains was 9.20 × 10^−4^ substitutions/site/year (95% HPD, 8.37 × 10^−4^–1.01 × 10^−3^). Cluster-specific estimates indicated substantial heterogeneity: Cluster 1 exhibited a rate of 3.37 × 10^−4^ (95% HPD, 2.17 × 10^−4^–4.56 × 10^−4^), Cluster 2 a rate of 9.54 × 10^−4^ (95% HPD, 7.66 × 10^−4^–1.15 × 10^−3^), and Cluster 3 a rate of 1.06 × 10^−3^ (95% HPD, 9.51 × 10^−4^–1.16 × 10^−3^). For the temporally balanced Cluster 3 dataset, the estimated rate was 9.77 × 10^−4^ substitutions/site/year (95% HPD: 8.60 × 10^−4^–1.10 × 10^−3^), which was similar in magnitude to the estimate from the original Cluster 3 dataset, with broadly overlapping 95% HPD intervals. The posterior point estimates were lowest for Cluster 1 and highest for Cluster 3. These differences were interpreted descriptively, considering the uncertainty represented by the 95% HPD intervals.

### 3.5. Phylogenetic Distance

**Figure 4:**
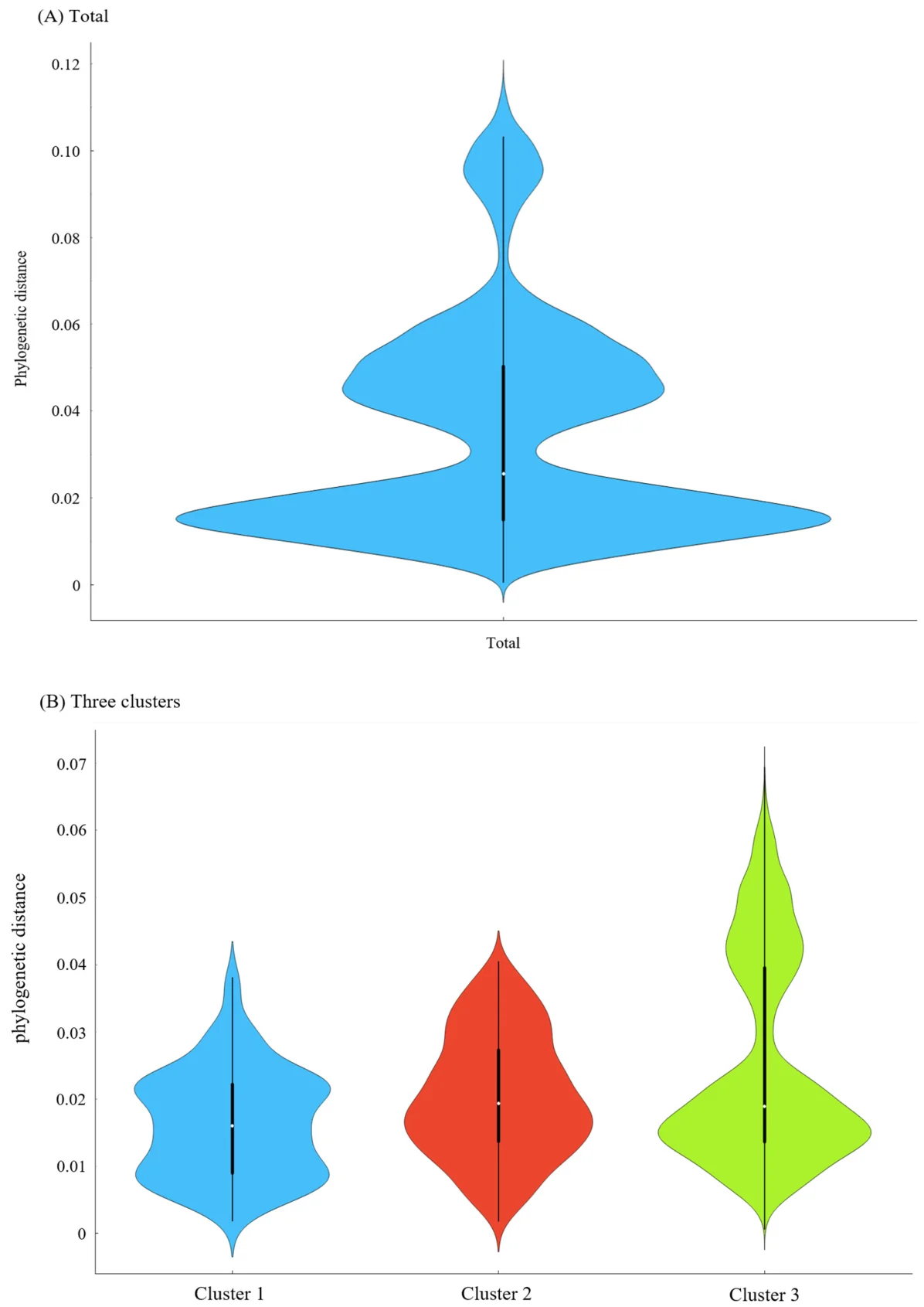
Phylogenetic distances of the HMPV-A *F* gene for all strains (**A**) and the three clusters (**B**). The *x*-axis indicates the clusters, and the *y*-axis indicates phylogenetic distance (substitutions per site). The white dot in the graph indicates the median. The distributions are presented for descriptive purposes only; pairwise patristic distances were not treated as independent observations for statistical testing.

Pairwise patristic distances were calculated from the ML phylogenetic trees to assess genetic diversity within the analyzed datasets, and the distributions were visualized using violin plots (Figure 4). For the complete HMPV-A dataset, the mean genetic distance was 0.036, and the median (interquartile range [IQR]) was 0.026 (0.015–0.050). When analyzed by cluster, Cluster 1 had the lowest mean distance (0.016), followed by Cluster 2 (0.020) and Cluster 3 (0.025). The median (IQR) values were 0.016 (0.009–0.022) for Cluster 1, 0.019 (0.014–0.027) for Cluster 2, and 0.019 (0.014–0.040) for Cluster 3. Descriptively, Cluster 1 had the lowest mean patristic distance, whereas Cluster 3 had the highest mean distance and the widest IQR. The distance distributions were interpreted descriptively without statistical significance testing. Together with the cluster-specific evolutionary-rate estimates, these results indicate that Cluster 1 had the lowest substitution rate and within-cluster phylogenetic diversity, whereas Clusters 2 and 3 displayed broader patristic-distance distributions. Cluster 3 had the highest mean distance and the widest IQR, consistent with greater genetic diversity than the other clusters (Supplementary Table S6).

### 3.6. Positive and Negative Selection Sites

To elucidate the selection pressures acting on the HMPV-A F protein, we analyzed its coding region to identify sites under positive and negative selection. In the complete dataset, MEME identified codons 188 and 271 as candidate positively selected sites, whereas FUBAR identified codon 528. FEL and SLAC did not identify any candidate positively selected codons, and no site was supported by all four methods.

To assess the potential influence of the predominance of Cluster 3, positive-selection analyses were repeated separately for each cluster. MEME identified codons 188 and 271 in Cluster 3, reproducing the two MEME signals observed in the complete dataset, whereas no candidate positively selected codons were detected in Clusters 1 or 2. The FUBAR signal at codon 528 was not reproduced in any cluster-specific analysis. In the temporally balanced Cluster 3 dataset, MEME continued to identify codon 271, whereas codon 188 was no longer detected; FUBAR identified codon 518 instead of codon 528 (Supplementary Table S12). Given the smaller numbers of sequences in Clusters 1 and 2, the absence of significant signals in these clusters was interpreted cautiously.

In contrast, in the complete dataset, 299 codons (approximately 55%) were consistently identified as being under negative selection by SLAC, FEL, and FUBAR. These negatively selected sites were widely distributed across the F-protein sequence (Supplementary Figure S2).

### 3.7. F Protein Sequence Identity

**Figure 5:**
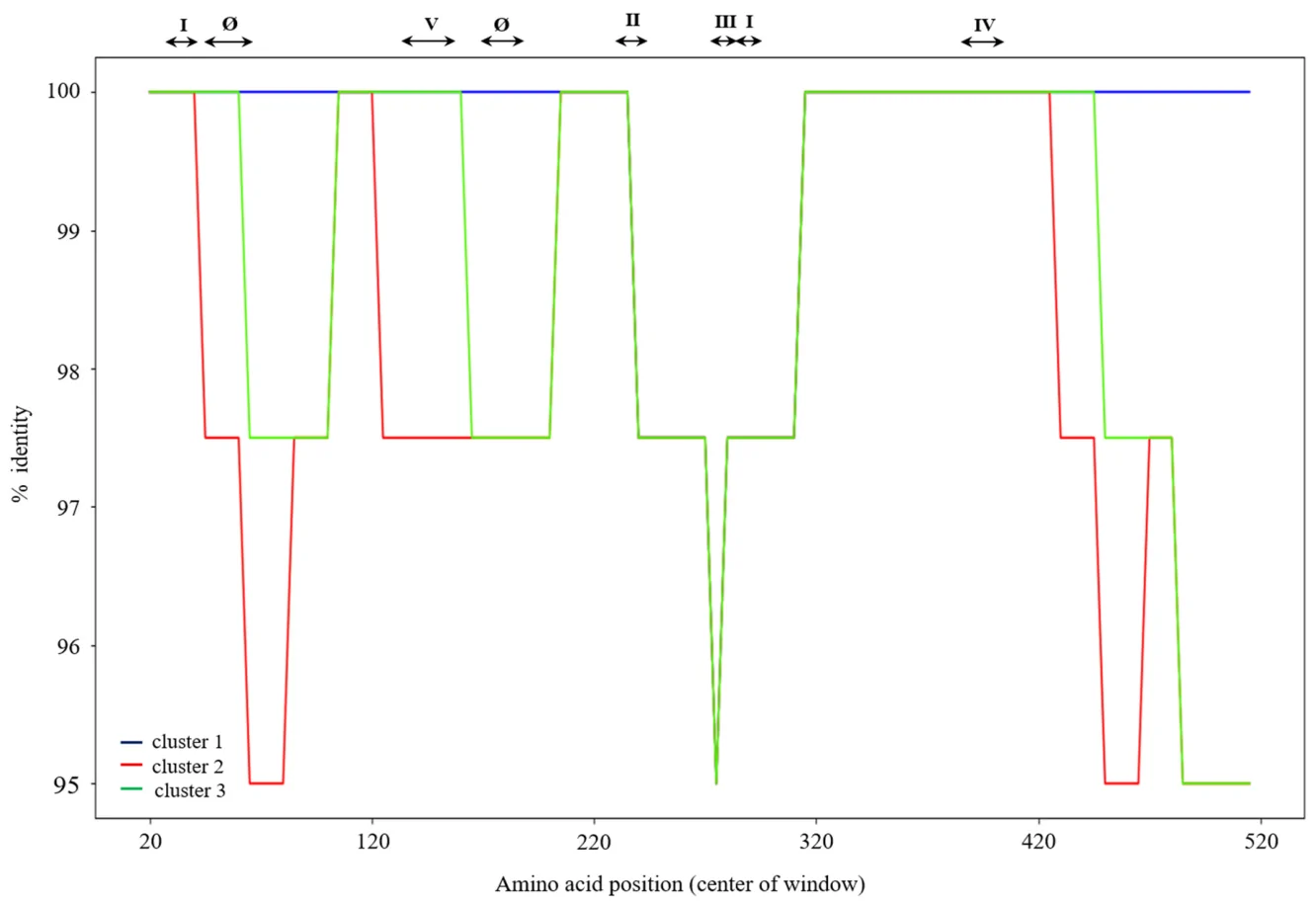
Sequence identity between HMPV-A F proteins and the HMPV reference strain. The plot compares the sequence identity of Cluster 1 (blue), Cluster 2 (red), and Cluster 3 (green) relative to the HMPV reference strain. The *x*-axis represents amino acid position, and the *y*-axis represents percentage sequence identity. Roman numerals (Ø and I–V) at the top indicate the approximate locations of neutralizing antibody-binding sites.

To evaluate HMPV-A F-protein sequence identity, sequences from Clusters 1, 2, and 3 were compared with the reference strain (Figure 5). Because the reference strain belongs to Cluster 1, the remaining 20 Cluster 1 sequences were used for the within-cluster comparison. SimPlot++ analysis showed that the mean percentage identity of each cluster relative to the reference strain exceeded 95%. All three clusters showed reduced identity in the central region spanning approximately amino acid positions 220–320. The identity profiles of the three clusters overlapped in this region, suggesting that they shared a region of relatively reduced sequence identity. Across the full length of the F protein, Cluster 1 exhibited the highest overall identity, with many regions reaching 100% identity. Clusters 2 and 3 also showed high similarity to the reference strain; however, Cluster 3 displayed identity values comparable to or higher than those of Cluster 2 across most regions. Whole-sequence identity, calculated by comparing each amino acid position with the reference strain, was 99.48% for Cluster 1, 97.98% for Cluster 2, and 98.37% for Cluster 3, indicating high overall sequence identity in all three clusters. However, because a gap was observed at codon 291 in one of the Cluster 3 strains (GenBank accession: EU_857608), sequence identity was calculated by treating that gap as an amino acid residue different from the reference sequence.

To further assess within-cluster amino acid diversity independently of the Cluster 1 reference strain, consensus-based and pairwise identity analyses were performed using the full-length F-protein sequences (Supplementary Table S13). Mean identity to the corresponding cluster-specific consensus was 99.8% for Cluster 1, 99.7% for Cluster 2, and 99.8% for Cluster 3, with a median of 99.8% in all three clusters. Mean within-cluster pairwise identity was 99.5%, 99.4%, and 99.6% for Clusters 1, 2, and 3, respectively, with a median of 99.6% in each cluster. These reference-independent analyses confirmed a high degree of amino acid conservation within all three clusters.

High identity was also maintained across the neutralizing antibody-binding sites (site Ø and sites I–V), ranging from 93.94% to 100%, with all three clusters showing comparable values. The greatest inter-cluster difference was observed at site Ø (residues 54–65), where Clusters 1 and 3 showed high identity (99.58% and 99.98%, respectively), whereas Cluster 2 exhibited a lower value (93.94%). These findings indicate that the F protein is generally well conserved across clusters, although subtle sequence-level differences are present (Supplementary Table S7). To further characterize these differences, we analyzed amino acid variation frequencies at each residue within the neutralizing antibody-binding sites (site Ø and sites I–V) and compared the predominant residues among the three clusters. The predominant residues at these sites were largely shared across clusters, and amino acid variation was observed only in a small subset of strains. Differences in predominant residues among clusters were identified at only two positions: residue 61 in site Ø and residue 143 in site V. At residue 61, alanine (A) was predominant in Clusters 1 and 3, whereas serine (S) was predominant in Cluster 2 (S: 72.73%; A: 27.27%). Similarly, at residue 143, lysine (K) was predominant in Clusters 1 and 3, whereas threonine (T) predominated in Cluster 2 (T: 68.18%; K: 31.82%). No differences in the predominant residue were observed among clusters at any other neutralizing antibody-binding site (Supplementary Table S8).

### 3.8. Structural Modeling and Conformational Epitope Mapping

**Figure 6:**
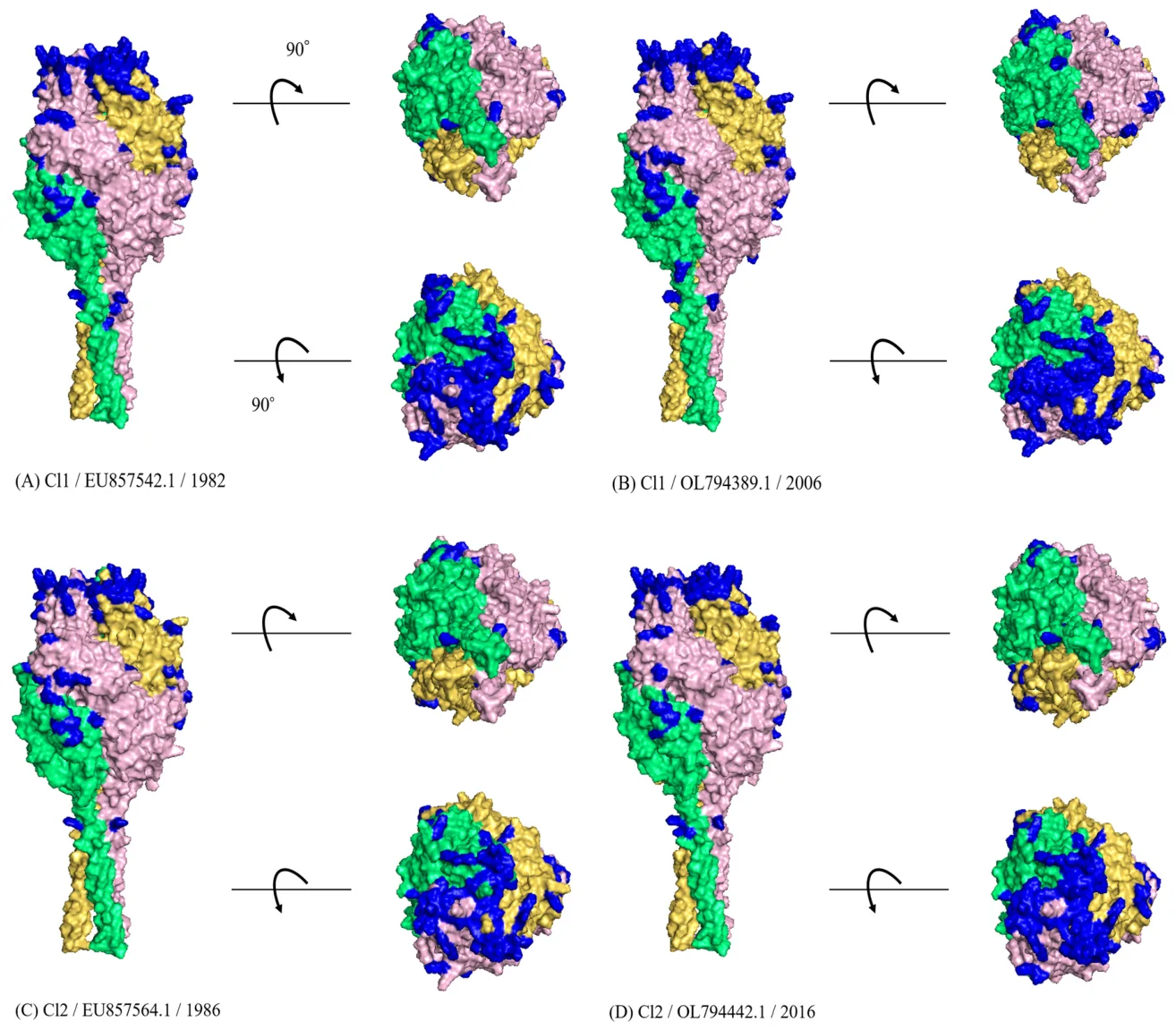

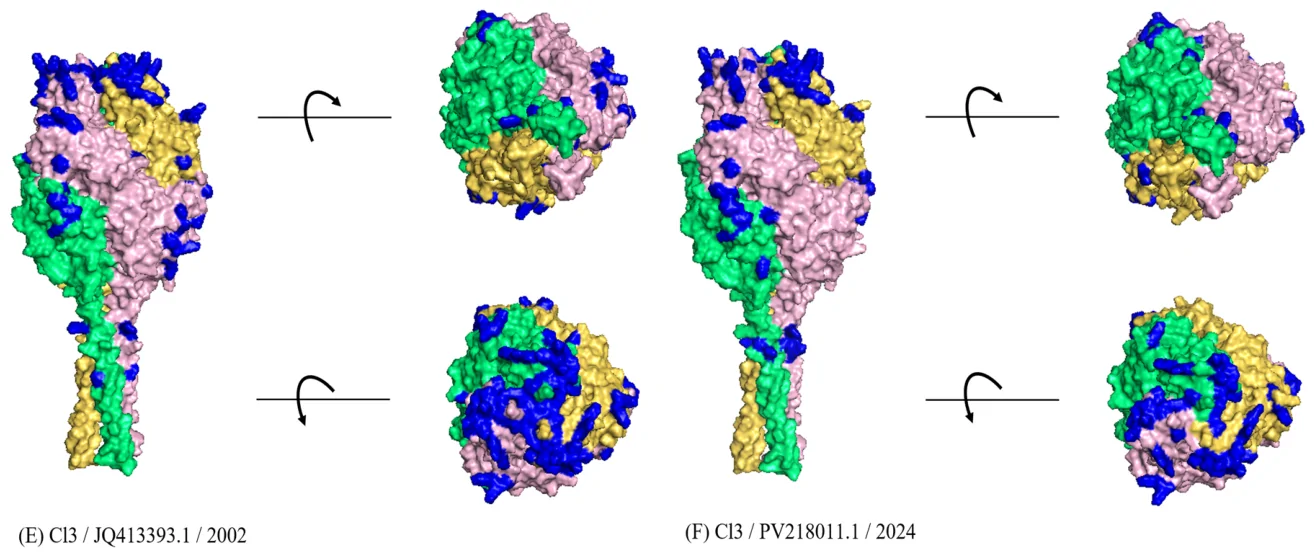
Representative structural models of the HMPV-A F protein. Each panel shows the predicted structure of a specific F protein: (**A**) the 1982 strain from Cluster 1 (EU857542.1), (**B**) the 2006 strain from Cluster 1 (OL794389.1), (**C**) the 1986 strain from Cluster 2 (EU857564.1), (**D**) the 2016 strain from Cluster 2 (OL794442.1), (**E**) the 2002 strain from Cluster 3 (JQ413393.1), and (**F**) the 2024 strain from Cluster 3 (PV218011.1). In each model, the three chains forming the trimer are colored light pink, yellow orange, and lime green, respectively. Predicted conformational B-cell epitopes are highlighted in blue. Cl denotes cluster.

**Figure 7:**
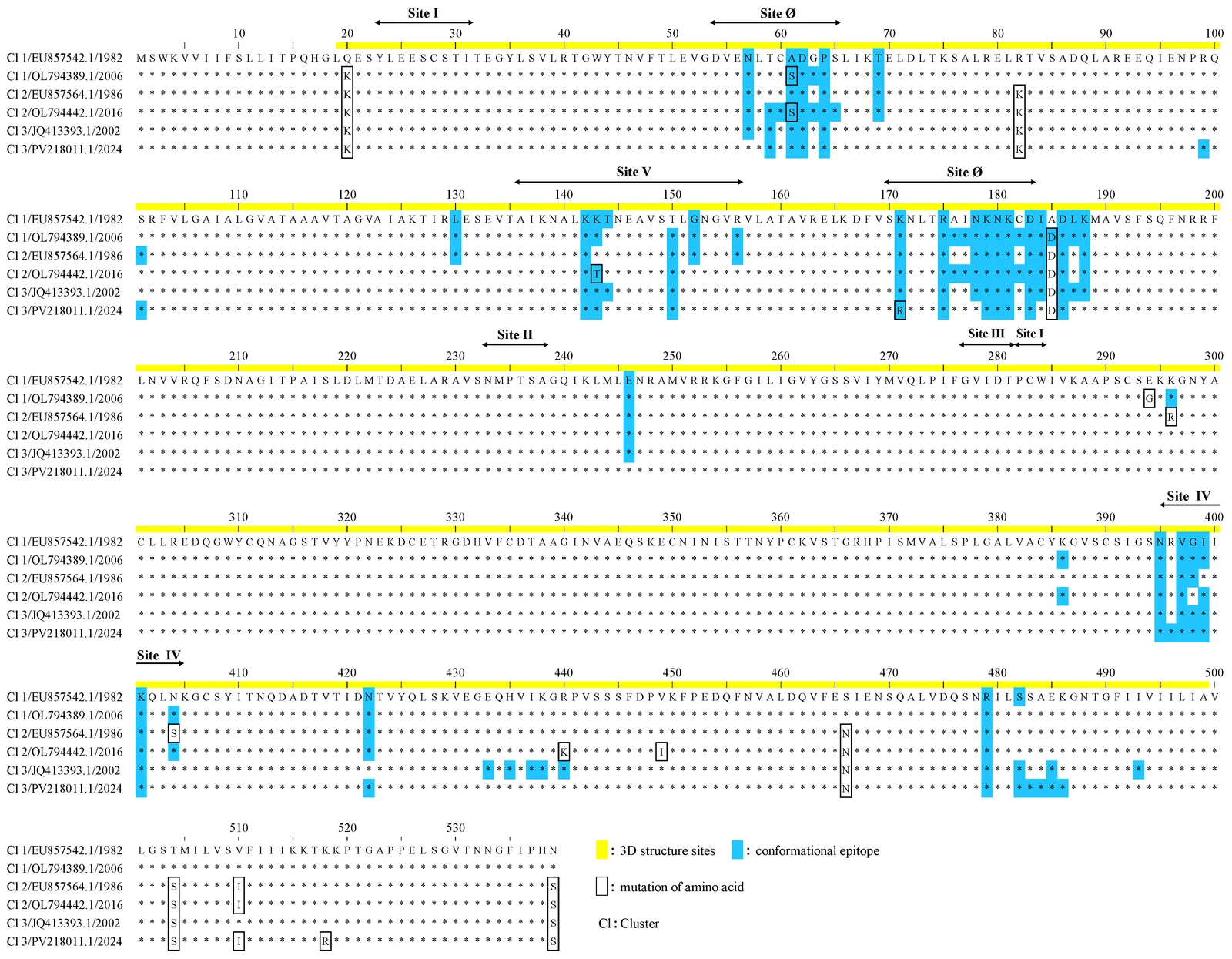
Amino acid sequence alignment of representative HMPV-A F proteins. The sequence region corresponding to the three-dimensional structural model is highlighted in yellow. Amino acid substitutions among the aligned strains are enclosed in black boxes. Predicted conformational B-cell epitope sites are highlighted in light blue. Amino acid positions are numbered according to the full-length 539-amino-acid F protein. The 1982 Cluster 1 strain EU857542.1 was used as the reference sequence. Asterisks (*) indicate amino acid residues identical to those of the reference sequence at the corresponding positions.

Three-dimensional models of the F protein were generated for representative HMPV-A strains, and predicted conformational B-cell epitopes were mapped onto their surfaces to compare their predicted spatial distributions (Figure 6A–F). The predicted trimeric structures consistently adopted a tree-like conformation comprising a globular head and a stalk region. Predicted conformational epitopes were predominantly located in the globular head region across all analyzed strains. A high degree of conservation was observed among Clusters 1, 2, and 3 in both amino acid sequence and predicted epitope distribution. Cα RMSD values calculated for pairwise comparisons of the six strains were 0.096 Å within Cluster 1, 0.247 Å within Cluster 2, and 0.238 Å within Cluster 3. Inter-cluster Cα RMSD values ranged from 0.041 to 0.268 Å. All values were low, indicating minimal divergence in the Cα backbone among the modeled structures. Visual superimposition also revealed no major structural differences, supporting a high degree of structural conservation among the six F-protein models. Although the predicted models were highly similar, specific amino acid substitutions were identified within predicted epitope sites (Figure 7). Some of these substitutions were located within experimentally defined neutralizing antibody-binding sites: A61S in the 2006 Cluster 1 strain (OL794389.1) and A61S and K143T in the 2016 Cluster 2 strain (OL794442.1). Although A61S and K143T occurred within experimentally defined neutralizing antibody-binding sites, no major differences in global backbone topology or overall predicted epitope-surface distribution were apparent among the six modeled representatives. These computational observations, however, do not establish unchanged antibody recognition or the functional neutrality of these substitutions. A comprehensive list of predicted epitope sites is provided in Supplementary Table S9, and detailed Cα RMSD values are provided in Supplementary Table S10.

## 4. Discussion

The present study demonstrates that HMPV-A *F* gene evolution is characterized by marked lineage-specific heterogeneity at the nucleotide level but strong conservation at the protein and predicted structural levels. Three well-supported phylogenetic clusters showed distinct temporal histories, evolutionary rates, phylogenetic diversity, and demographic patterns (Figure 1, Figure 3, and Figure 4; Supplementary Figure S5). Cluster 1 was the earliest-diverging lineage and had the lowest estimated substitution rate and within-cluster diversity, whereas Cluster 3 had the highest estimated rate, the greatest mean patristic distance, and the most pronounced recent demographic fluctuations. In contrast, F protein sequence identity remained high across all clusters, negative selection was extensive, and major neutralizing antibody-binding regions and predicted F protein structures were largely conserved (Figure 5, Figure 6 and Figure 7; Supplementary Figure S2). Together, these observations indicate that substantial *F* gene diversification can occur without corresponding large-scale diversification of the F protein.

First, the time-scaled phylogeny supported a stepwise history of HMPV-A *F* gene diversification (Figure 1; Supplementary Figure S5). Cluster 1 diverged from the common ancestor of Clusters 2 and 3 around 1927, followed by diversification of the common ancestor of Clusters 2 and 3 around 1972 and subsequent diversification of Clusters 2 and 3 around 1974 and 1980, respectively. The minimum-spanning network provided a complementary visualization of sequence relationships and showed patterns broadly consistent with these phylogenetic assignments, with Clusters 1 and 2 appearing more compact and Cluster 3 distributed more broadly across the network (Figure 2). The three operational clusters largely recapitulated the available HMPV-A lineage assignments: Cluster 1 corresponded to A1, Cluster 2 to A2.1, and Cluster 3 encompassed the A2.2-derived lineages. The value of this grouping is therefore not the introduction of a new nomenclature, but the use of a consistent *F*-gene phylogenetic framework for comparing lineage-level differences in evolutionary rate, genetic diversity, demographic history, sequence conservation, and structural features. Because Cluster 3 combines A2.2, A2.2.1, and A2.2.2, estimates for this cluster should be interpreted as group-level summaries and not necessarily as properties shared by each individual A2.2-derived sublineage.

Second, the major nodes defining Clusters 1–3 were strongly supported, with posterior probabilities of 1.00 (Supplementary Figure S5). Several branch-specific random-local-clock parameters showed low ESS values, indicating limited mixing and resolution of individual local rate shifts. In contrast, the global posterior quantities reported in this study had ESS values >200. However, satisfactory ESS values for these marginal summaries do not establish complete convergence of all components of the joint posterior distribution. We therefore did not infer branch-specific acceleration or deceleration or the precise number, location, or magnitude of local rate shifts. Divergence-time estimates should accordingly be interpreted together with their 95% HPD intervals rather than as exact historical dates. The limited number of sequences from the 1980s and 1990s, together with the small sizes of Clusters 1 and 2, reduces the temporal information available for deep divergence-time and demographic inference. The reported 95% HPD intervals therefore represent posterior uncertainty conditional on the fitted models and do not encompass all uncertainty associated with historical undersampling and uneven sequence availability. Moreover, PARNAS retained 97.17% of the phylogenetic diversity of the 930 non-redundant sequences while reducing the analytical dataset to 500 representatives. Nevertheless, the resulting dataset remained markedly unbalanced, with 435 sequences assigned to Cluster 3. The predominance of Cluster 3 in the selected dataset should be interpreted cautiously because PARNAS preserves phylogenetic representativeness rather than population frequencies, and publicly available sequences are unevenly sampled across countries and years. The cluster composition is therefore most appropriately viewed as a property of the analyzed sequence dataset.

Bayesian skyline analyses indicated distinct demographic histories among the three clusters (Figure 3 and Supplementary Figure S4). Cluster 3 showed the most pronounced temporal variation in the original dataset. However, the sensitivity analysis using the temporally balanced Cluster 3 dataset showed that the broad increase in inferred effective population size from the late 2000s onward and the recent increase near the end of the sampling period were retained, whereas shorter-term peaks and declines were attenuated and their precise timing differed (Supplementary Figure S4). These findings suggest that the broad long-term demographic tendency was not solely driven by temporal sampling intensity, whereas fine-scale skyline features were sensitive to sampling composition. Accordingly, individual peaks and declines should not be interpreted as direct reconstructions of epidemic activity. Bayesian skyline plots reflect changes in genealogically inferred effective population size rather than case incidence, and estimates near the temporal boundaries of the dataset remain particularly uncertain.

Furthermore, the evolutionary-rate estimates and patristic-distance distributions showed variation among the three clusters. Cluster 1 had the lowest estimated substitution rate and mean patristic distance, Cluster 2 showed intermediate values, and Cluster 3 had the highest estimates for both measures (Figure 4; Supplementary Table S6). However, these differences should be interpreted descriptively, because MCMC posterior samples do not represent independent biological observations, and pairwise patristic distances are not independent. The overall evolutionary rate of 9.20 × 10^−4^ substitutions/site/year was of the same order of magnitude as previous estimates for HMPV *F* genes, including HMPV-B [14,19]. These findings suggest that a single pooled evolutionary-rate estimate may obscure variation among phylogenetic lineages, even for a relatively conserved viral gene.

Despite this nucleotide-level heterogeneity, the F protein was strongly conserved (Figure 5 and Supplementary Figure S2). The original reference-based analysis showed high whole-protein identity across all three clusters, and the reference-independent consensus-based and pairwise analyses likewise demonstrated high within-cluster F-protein identity. Mean within-cluster pairwise identities were 99.5%, 99.4%, and 99.6% for Clusters 1, 2, and 3, respectively (Supplementary Table S13). These complementary analyses indicate that the observed protein conservation was not solely dependent on the use of the Cluster 1 strain NC_039199.1 as a common reference. The reference-based and within-cluster analyses address different aspects of sequence variation: the former provides a common benchmark across phylogenetic groups, whereas the latter directly characterizes diversity within each cluster. In addition, 299 of 539 codons (approximately 55%) were consistently identified as being under negative selection by SLAC, FEL, and FUBAR (Supplementary Figure S2). This extensive purifying selection is consistent with the multiple functional constraints imposed on the F protein, including proteolytic processing, trimer formation, membrane-fusion activity, and maintenance of conformational surfaces recognized by neutralizing antibodies [13,14,15]. Thus, much of the accumulated nucleotide variation appears not to have translated into broad amino acid diversification. In addition, evidence for positive selection was comparatively weak. FUBAR identified codon 528, whereas MEME identified codons 188 and 271, but no codon was supported as being under positive selection by all four methods. Evidence for diversifying selection was limited and method-dependent rather than absent (Supplementary Table S12). The cluster-stratified analysis again identified codons 188 and 271 by MEME in Cluster 3, indicating that these signals were detectable within Cluster 3 rather than arising solely from analysis of the pooled dataset. However, in the temporally balanced Cluster 3 dataset, codon 271 remained detectable by MEME whereas codon 188 was no longer detected, and FUBAR identified codon 518 rather than codon 528. These results indicate that codon 271 was the most consistently detected candidate signal, whereas the other site-specific signals were more sensitive to dataset composition. No significant positively selected sites were detected in Clusters 1 or 2; however, given their substantially smaller sample sizes, this should not be interpreted as evidence that diversifying selection is absent from these lineages. Overall, the candidate sites identified here should be regarded as method- and dataset-dependent signals requiring further investigation. Against this background, the extensive distribution of negatively selected sites remains consistent with purifying selection as the predominant selective pressure acting on the HMPV-A F protein.

Major neutralizing antibody-binding regions were also highly conserved (Figure 5; Supplementary Table S7) [16,17,18]. Differences in the predominant amino acid among clusters were identified at only two positions: residue 61 in site Ø and residue 143 in site V (Supplementary Table S8). A61S occurred in both Cluster 1 and Cluster 2 representatives (Figure 7) and was enriched in Cluster 2 (Supplementary Table S8), indicating that it is not a strictly cluster-specific marker. K143T predominated among Cluster 2 sequences (Supplementary Table S8) and was present in the later Cluster 2 representative (Figure 7), but the present phylogenetic analysis does not establish precisely when or how this substitution arose. Notably, variation at residue 143 was also observed in our previous HMPV-B analysis [19], making this position of particular interest for experimental evaluation of antibody binding and fusion-related function.

Structural modeling was consistent with the sequence-based evidence for conservation (Figure 6). The six representative F proteins showed very low intra- and inter-cluster Cα RMSD values, with all pairwise values ≤0.268 Å, and predicted conformational B-cell epitopes occupied similar regions of the modeled protein surface (Figure 6; Supplementary Table S10). A61S and K143T should be regarded as candidate substitutions requiring experimental evaluation (Figure 7). Although no major differences in global backbone topology or overall predicted epitope-surface distribution were apparent among the modeled representatives, Cα RMSD and visual structural comparison cannot exclude changes in local side-chain orientation, surface charge distribution, glycosylation, conformational dynamics, or antibody-binding affinity [16,17,18]. Direct binding, neutralization, and fusion assays will be required to determine their biological significance.

The present results broadly parallel our previous findings for HMPV-B, in which continued nucleotide evolution and lineage-specific demographic differences occurred against a background of pronounced F-protein conservation [19]. Taken together, the HMPV-A and HMPV-B analyses support the concept that strong functional constraint of the F protein is a shared feature of the major HMPV genetic groups. For HMPV-A, the predominance of purifying selection together with only limited method- and dataset-dependent candidate signals of diversifying selection supports the overall functional constraint of the F protein (Supplementary Figure S2; Supplementary Table S12). These features are favorable for F-directed vaccine and antibody-based strategies, while the recurrent or lineage-enriched substitutions identified here underscore the need for continued residue-level genomic surveillance [4,16,19].

Finally, several limitations should be acknowledged. First, publicly available HMPV sequences are unevenly distributed across geographic regions, collection years, and lineages, and PARNAS-based down-selection cannot fully account for biases inherent in the underlying sequence database. Second, reducing 930 non-redundant sequences to 500 representatives, although retaining 97.17% of phylogenetic diversity, may have reduced representation of rare or recently sampled variants. Third, the analysis was restricted to the *F* gene and therefore cannot capture evolutionary changes elsewhere in the viral genome. Although the operational clusters corresponded closely to the available Nextclade assignments in this dataset, they were used for analytical purposes and should not be regarded as independent nomenclatural entities. Fourth, the structural analysis was based on only two selected representatives per cluster. Therefore, the observed similarity should be interpreted as conservation of the global backbone topology among the six modeled representatives rather than as evidence of structural invariance throughout each cluster. Local conformational or epitope-surface differences associated with within-cluster sequence variation may not be captured by the present models. In addition, these computational predictions cannot substitute for experimental measurements of antibody binding, neutralization, membrane fusion, or protein stability. Finally, divergence-time, substitution-rate, and Bayesian skyline estimates remain dependent on sequence sampling and model assumptions. In particular, the limited number of sequences from the 1980s and 1990s and the small sizes of Clusters 1 and 2 reduce the temporal information available for divergence-time, evolutionary-rate, and demographic inference. Accordingly, the reported 95% HPD intervals should be interpreted as model-conditional posterior uncertainty and do not capture all uncertainty arising from historical undersampling. These limitations warrant caution in extrapolating the present phylogenetic and structural findings to population prevalence or antigenic phenotype.

## 5. Conclusions

The HMPV-A *F* gene segregated into three well-supported phylogenetic clusters with different estimated evolutionary rates, genetic diversity, and demographic histories. Cluster 3 showed the highest estimated substitution rate and mean phylogenetic diversity and the most pronounced recent demographic fluctuations, whereas Cluster 1 evolved more slowly and remained comparatively less diverse. Despite these lineage-specific nucleotide-level differences, the F protein was highly conserved, with extensive purifying selection together with limited, method-specific candidate signals of diversifying selection, and preservation of major neutralizing antibody-binding regions and predicted protein structures. A61S and K143T represent naturally occurring substitutions within neutralizing antibody-binding sites that merit direct functional evaluation rather than evidence of antigenic escape. Overall, these findings support the HMPV F protein as a broadly conserved target for vaccine and antibody-based interventions while emphasizing the value of continued genomic surveillance and experimental assessment of lineage-enriched amino acid variants.

## Data Availability

The original contributions presented in this study are included in the article/Supplementary Material. Further inquiries can be directed to the corresponding authors.

## Supplementary Materials

The following supporting information can be downloaded at: https://www.mdpi.com/article/10.3390/microorganisms14102386/s1, Figure S1, Temporal distribution of HMPV-A strains by collection year; Figure S2, Sites under negative selection in the HMPV-A F protein; Figure S3, Cluster-specific consensus sequences and representative-strain sequences across the core regions of neutralizing antibody-binding sites; Figure S4, Comparison of Bayesian skyline trajectories and temporal lineage composition between the original and temporally balanced Cluster 3 datasets; Figure S5, Time-scaled maximum clade credibility tree of HMPV-A *F*-gene sequences showing posterior probability support values; Table S1, HMPV-A sequences excluded during PARNAS-based phylogenetic down-selection; Table S2, List of HMPV-A strains and associated metadata used in this study; Table S3, Parameter settings for the BMCMC analyses; Table S4, Summary statistics and effective sample size (ESS) values for each parameter from the BEAST analyses; Table S5, Detailed temporal and geographic distribution of HMPV-A *F*-gene sequences in the original and temporally balanced datasets; Table S6, Phylogenetic distances of the HMPV-A *F* gene for all strains and the three clusters; Table S7, Amino acid identity of neutralizing antibody-binding sites between the HMPV-A reference strain and the three clusters; Table S8, Site-specific amino acid variation frequencies and predominant residues within neutralizing antibody-binding sites; Table S9, Predicted conformational B-cell epitopes on the HMPV-A F protein; Table S10, Pairwise Cα RMSD values among the six representative HMPV-A F-protein structures; Table S11, Marginal likelihood estimates obtained by path-sampling/stepping-stone analysis for model selection in the HMPV-A *F*-gene analyses; Table S12, Cluster-stratified and sampling-sensitivity analyses of candidate positively selected codons in the HMPV-A *F* gene; Table S13, Consensus-based and pairwise amino acid identity summaries of the HMPV-A F protein.

## References

[1] van den Hoogen B.G., de Jong J.C., Groen J., Kuiken T., de Groot R., Fouchier R.A., Osterhaus A.D. (2001). A newly discovered human pneumovirus isolated from young children with respiratory tract disease. Nat. Med..

[2] Wang X., Li Y., Deloria-Knoll M., Madhi S.A., Cohen C., Ali A., Basnet S., Bassat Q., Brooks W.A., Chittaganpitch M. (2021). Global burden of acute lower respiratory infection associated with human metapneumovirus in children under 5 years in 2018: A systematic review and modelling study. Lancet Glob. Health.

[3] Lefebvre A., Manoha C., Bour J.B., Abbas R., Fournel I., Tiv M., Pothier P., Astruc K., Aho-Glele L.S. (2016). Human metapneumovirus in patients hospitalized with acute respiratory infections: A meta-analysis. J. Clin. Virol..

[4] Guo L., Li L., Liu L., Zhang T., Sun M. (2023). Neutralising antibodies against human metapneumovirus. Lancet Microbe.

[5] van den Hoogen B.G., Herfst S., Sprong L., Cane P.A., Forleo-Neto E., de Swart R.L., Osterhaus A.D., Fouchier R.A. (2004). Antigenic and genetic variability of human metapneumoviruses. Emerg. Infect. Dis..

[6] Biacchesi S., Skiadopoulos M.H., Boivin G., Hanson C.T., Murphy B.R., Collins P.L., Buchholz U.J. (2003). Genetic diversity between human metapneumovirus subgroups. Virology.

[7] Heim C.J., van den Hoogen B.G., Dutch R.E. (2025). Human metapneumovirus: Understanding the molecular mechanisms and pathology of infection. J. Virol..

[8] Gaunt E.R., Jansen R.R., Poovorawan Y., Templeton K.E., Toms G.L., Simmonds P. (2011). Molecular epidemiology and evolution of human respiratory syncytial virus and human metapneumovirus. PLoS ONE.

[9] Nao N., Saikusa M., Sato K., Sekizuka T., Usuku S., Tanaka N., Nishimura H., Takeda M. (2020). Recent Molecular Evolution of Human Metapneumovirus (HMPV): Subdivision of HMPV A2b Strains. Microorganisms.

[10] Saikusa M., Kawakami C., Nao N., Takeda M., Usuku S., Sasao T., Nishimoto K., Toyozawa T. (2017). 180-Nucleotide Duplication in the G Gene of Human metapneumovirus A2b Subgroup Strains Circulating in Yokohama City, Japan, since 2014. Front. Microbiol..

[11] Saikusa M., Nao N., Kawakami C., Usuku S., Sasao T., Toyozawa T., Takeda M., Okubo I. (2017). A novel 111-nucleotide duplication in the G gene of human metapneumovirus. Microbiol. Immunol..

[12] Munoz-Escalante J.C., Mata-Moreno G., Rivera-Alfaro G., Noyola D.E. (2022). Global Extension and Predominance of Human Metapneumovirus A2 Genotype with Partial G Gene Duplication. Viruses.

[13] Schowalter R.M., Smith S.E., Dutch R.E. (2006). Characterization of human metapneumovirus F protein-promoted membrane fusion: Critical roles for proteolytic processing and low pH. J. Virol..

[14] Yang C.F., Wang C.K., Tollefson S.J., Piyaratna R., Lintao L.D., Chu M., Liem A., Mark M., Spaete R.R., Crowe J.E. (2009). Genetic diversity and evolution of human metapneumovirus fusion protein over twenty years. Virol. J..

[15] Skiadopoulos M.H., Biacchesi S., Buchholz U.J., Amaro-Carambot E., Surman S.R., Collins P.L., Murphy B.R. (2006). Individual contributions of the human metapneumovirus F, G, and SH surface glycoproteins to the induction of neutralizing antibodies and protective immunity. Virology.

[16] Huang J., Diaz D., Mousa J.J. (2019). Antibody Epitopes of Pneumovirus Fusion Proteins. Front. Immunol..

[17] Battles M.B., Mas V., Olmedillas E., Cano O., Vazquez M., Rodriguez L., Melero J.A., McLellan J.S. (2017). Structure and immunogenicity of pre-fusion-stabilized human metapneumovirus F glycoprotein. Nat. Commun..

[18] Wen X., Krause J.C., Leser G.P., Cox R.G., Lamb R.A., Williams J.V., Crowe J.E., Jardetzky T.S. (2012). Structure of the human metapneumovirus fusion protein with neutralizing antibody identifies a pneumovirus antigenic site. Nat. Struct. Mol. Biol..

[19] Shirai T., Mizukoshi F., Sada M., Shirato K., Saraya T., Ishii H., Kimura R., Sugai T., Ryo A., Kimura H. (2026). Molecular Evolution of the Fusion (F) Genes in Human Metapneumovirus Genotype B. Microorganisms.

[20] Katoh K., Standley D.M. (2013). MAFFT multiple sequence alignment software version 7: Improvements in performance and usability. Mol. Biol. Evol..

[21] Aksamentov I., Roemer C., Hodcroft E.B., Neher R.A. (2021). Nextclade: Clade assignment, mutation calling and quality control for viral genomes. J. Open Source Softw..

[22] Wong T.K.F., Ly-Trong N., Ren H., Demotte P., Banos H., Roger A.J., Susko E., Bielow C., De Maio N., Goldman N. (2026). IQ-TREE 3: Phylogenomic inference software using complex evolutionary models. Mol. Biol. Evol..

[23] Markin A., Wagle S., Grover S., Vincent Baker A.L., Eulenstein O., Anderson T.K. (2023). PARNAS: Objectively Selecting the Most Representative Taxa on a Phylogeny. Syst. Biol..

[24] Bouckaert R., Vaughan T.G., Barido-Sottani J., Duchene S., Fourment M., Gavryushkina A., Heled J., Jones G., Kuhnert D., De Maio N. (2019). BEAST 2.5: An advanced software platform for Bayesian evolutionary analysis. PLoS Comput. Biol..

[25] Darriba D., Taboada G.L., Doallo R., Posada D. (2012). jModelTest 2: More models, new heuristics and parallel computing. Nat. Methods.

[26] Rambaut A., Drummond A.J., Xie D., Baele G., Suchard M.A. (2018). Posterior Summarization in Bayesian Phylogenetics Using Tracer 1.7. Syst. Biol..

[27] Excoffier L., Smouse P.E. (1994). Using allele frequencies and geographic subdivision to reconstruct gene trees within a species: Molecular variance parsimony. Genetics.

[28] Rozas J., Ferrer-Mata A., Sanchez-DelBarrio J.C., Guirao-Rico S., Librado P., Ramos-Onsins S.E., Sanchez-Gracia A. (2017). DnaSP 6: DNA Sequence Polymorphism Analysis of Large Data Sets. Mol. Biol. Evol..

[29] Leigh J.W., Bryant D. (2015). popart: Full-feature software for haplotype network construction. Methods Ecol. Evol..

[30] Kumar S., Stecher G., Tamura K. (2016). MEGA7: Molecular Evolutionary Genetics Analysis Version 7.0 for Bigger Datasets. Mol. Biol. Evol..

[31] Fourment M., Gibbs M.J. (2006). PATRISTIC: A program for calculating patristic distances and graphically comparing the components of genetic change. BMC Evol. Biol..

[32] Demšar J., Curk T., Erjavec A., Gorup Č., Hočevar T., Milutinovič M., Možina M., Polajnar M., Toplak M., Starič A. (2013). Orange: Data mining toolbox in python. J. Mach. Learn. Res..

[33] Weaver S., Shank S.D., Spielman S.J., Li M., Muse S.V., Kosakovsky Pond S.L. (2018). Datamonkey 2.0: A Modern Web Application for Characterizing Selective and Other Evolutionary Processes. Mol. Biol. Evol..

[34] Kosakovsky Pond S.L., Frost S.D. (2005). Not so different after all: A comparison of methods for detecting amino acid sites under selection. Mol. Biol. Evol..

[35] Murrell B., Moola S., Mabona A., Weighill T., Sheward D., Kosakovsky Pond S.L., Scheffler K. (2013). FUBAR: A fast, unconstrained bayesian approximation for inferring selection. Mol. Biol. Evol..

[36] Murrell B., Wertheim J.O., Moola S., Weighill T., Scheffler K., Kosakovsky Pond S.L. (2012). Detecting individual sites subject to episodic diversifying selection. PLoS Genet..

[37] Samson S., Lord E., Makarenkov V. (2022). SimPlot++: A Python application for representing sequence similarity and detecting recombination. Bioinformatics.

[38] Banerjee A., Huang J., Rush S.A., Murray J., Gingerich A.D., Royer F., Hsieh C.L., Tripp R.A., McLellan J.S., Mousa J.J. (2022). Structural basis for ultrapotent antibody-mediated neutralization of human metapneumovirus. Proc. Natl. Acad. Sci. USA.

[39] Mirdita M., Schutze K., Moriwaki Y., Heo L., Ovchinnikov S., Steinegger M. (2022). ColabFold: Making protein folding accessible to all. Nat. Methods.

[40] Schrödinger L. (2024). The PyMOL Molecular Graphics System.

[41] Hoie M.H., Gade F.S., Johansen J.M., Wurtzen C., Winther O., Nielsen M., Marcatili P. (2024). DiscoTope-3.0: Improved B-cell epitope prediction using inverse folding latent representations. Front. Immunol..

[42] Ponomarenko J., Bui H.H., Li W., Fusseder N., Bourne P.E., Sette A., Peters B. (2008). ElliPro: A new structure-based tool for the prediction of antibody epitopes. BMC Bioinform..

[43] Zhou C., Chen Z., Zhang L., Yan D., Mao T., Tang K., Qiu T., Cao Z. (2019). SEPPA 3.0-enhanced spatial epitope prediction enabling glycoprotein antigens. Nucleic Acids Res..

[44] Shashkova T.I., Umerenkov D., Salnikov M., Strashnov P.V., Konstantinova A.V., Lebed I., Shcherbinin D.N., Asatryan M.N., Kardymon O.L., Ivanisenko N.V. (2022). SEMA: Antigen B-cell conformational epitope prediction using deep transfer learning. Front. Immunol..

