# Supplementary material for: Molecular Evolution of the HMPV-A Fusion Gene Reveals Lineage-Specific Diversification and Structural Conservation
Source: Microorganisms. 2026 Oct 18;14(10):2386. doi: 10.3390/microorganisms14102386 (PMC13707938; doi:10.3390/microorganisms14102386)
Supplement: Supplementary file 1 [file microorganisms-14-02386-s001.zip › SupplementaryFigures_HMPVA_F.pdf]

Supplementary data

Molecular Evolution of the HMPV-A *Fusion* Gene Reveals Lineage-Specific Diversification and Structural Conservation

Kazuhiko Harada [1,2], Fuminori Mizukoshi [3], Tatsuya Shirai [4], Ryusuke Kimura [5], Koo Nagasawa [6], Kazuto Ito [7,8], Isao Kurosawa [7], Kazuya Shirato [9], Akihide Ryo [3], Hirokazu Kimura [1,8*]

[1] Department of Health Science, Gunma Paz University Graduate School, Takasaki-shi 370-0006, Gunma, Japan; k_harada@bishinkai.or.jp (K.H.); h-kimura@paz.ac.jp (H.K.)
[2] Department of Clinical Medical Laboratory, Medical Corporation Bishinkai, Kurosawa Hospital, Takasaki-shi 370-1203, Gunma, Japan
[3] Department of Bioinformatics and Integrative Omics, National Institute of Infectious Diseases, Japan Institute for Health Security, Musashimurayama-shi 208-0011, Tokyo, Japan; mizukoshi.f@jihs.go.jp (F.M.); ryo.a@jihs.go.jp (A.R.)
[4] Department of Respiratory Medicine, Faculty of Medicine, Kyorin University, Mitaka-shi 181-8611, Tokyo, Japan; tatsuya-shirai@ks.kyorin-u.ac.jp (T.S.)
[5] Department of Health Science, Gunma Prefectural Institute of Public Health and Environmental Sciences, Maebashi-shi, Gunma 371-0052, Gunma, Japan; kimura-r56@pref.gunma.lg.jp (R.K.)
[6] Department of Pediatrics, Graduate School of Medical Science, Chiba University, Chiba-shi 260-8670, Japan; k-nagasawa@chiba-u.jp (K.N.)
[7] Department of Urology, Medical Corporation Bishinkai, Kurosawa Hospital, Takasaki-shi 370-1203, Gunma, Japan; k_ito@bishinkai.or.jp (K.I.); i_kurosawa@bishinkai.or.jp (I.K.)
[8] Advanced Medical Science Research Center, Gunma Paz University, Takasaki-shi 370-0006, Gunma, Japan
[9] Department of Respiratory Viruses, National Institute of Infectious Diseases, Japan Institute for Health Security, Musashimurayama-shi 208-0011, Tokyo, Japan; shirato.k@jihs.go.jp (K.S.)
* Correspondence: h-kimura@paz.ac.jp; Tel.: +81-27-365-3366 (H.K.)

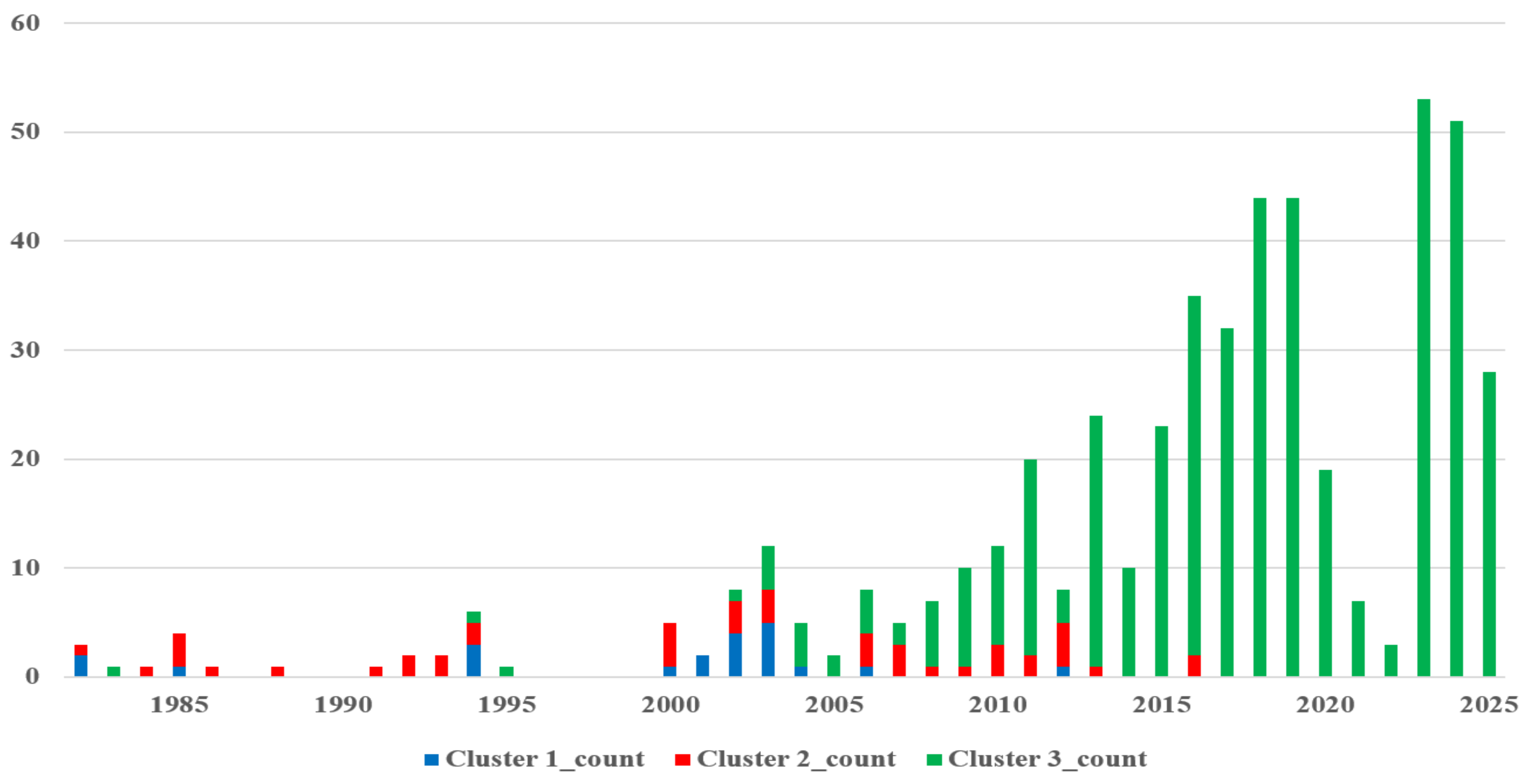

Figure S1. Temporal distribution of HMPV-A strains by collection year. The *x*-axis represents the collection year, and the *y*-axis indicates the number of strains. The bars in the bar chart represent the clusters: blue for Cluster 1, red for Cluster 2, and green for Cluster 3.

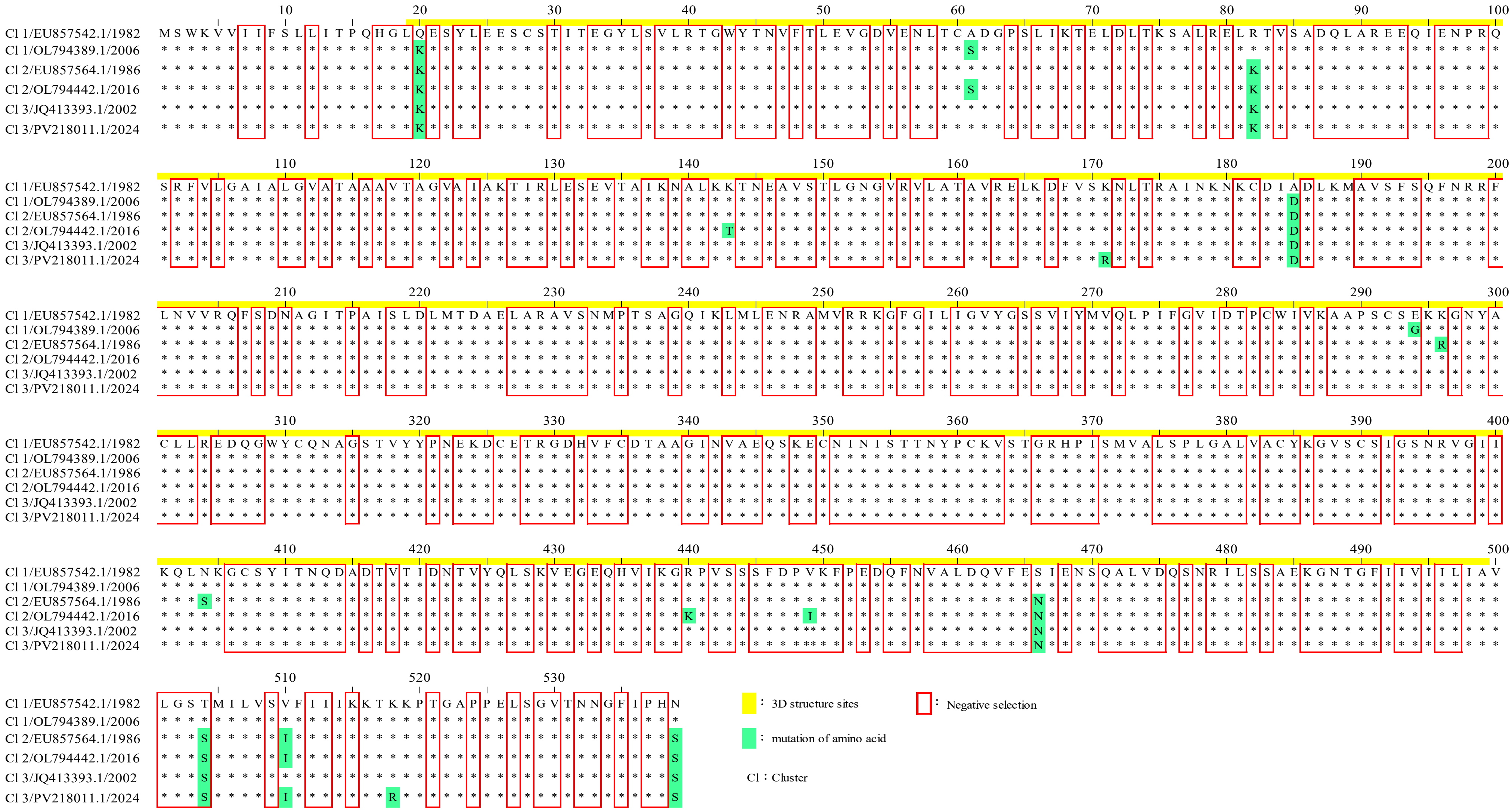

Figure S2. Sites under negative selection in the HMPV-A F protein. The sequence region corresponding to the 3D structural model is highlighted in yellow. Sites under negative selection are indicated by red boxes. Amino acid substitution sites among the aligned strains are highlighted in light green. The reference strain for the linear alignment was the oldest strain in the dataset, Cl1/EU857542.1/1982 (GenBank accession: EU857542.1). Asterisks (*) indicate positions where the amino acid is identical to the reference sequence.

Site Ø

residue number	54	55	56	57	58	59	60	61	62	63	64	65	170	171	172	173	174	175	176	177	178	179	180	181	182	183
Cl 1 consensus sequence	D	V	E	N	L	T	C	A	D	G	P	S	S	K	N	L	T	R	A	I	N	K	N	K	C	D
Cl 1/EU857542.1/1982	*	*	*	*	*	*	*	*	*	*	*	*	*	*	*	*	*	*	*	*	*	*	*	*	*	*
Cl 1/OL794389.1/2006	*	*	*	*	*	*	*	S	*	*	*	*	*	*	*	*	*	*	*	*	*	*	*	*	*	*
Cl 2 consensus sequence	D	V	E	N	L	T	C	S	D	G	P	S	S	K	N	L	T	R	A	I	N	K	N	K	C	D
Cl 2/EU857564.1/1986	*	*	*	*	*	*	*	A	*	*	*	*	*	*	*	*	*	*	*	*	*	*	*	*	*	*
Cl 2/OL794442.1/2016	*	*	*	*	*	*	*	*	*	*	*	*	*	*	*	*	*	*	*	*	*	*	*	*	*	*
Cl 3 consensus sequence	D	V	E	N	L	T	C	A	D	G	P	S	S	K	N	L	T	R	A	I	N	K	N	K	C	D
Cl 3/JQ413393.1/2002	*	*	*	*	*	*	*	*	*	*	*	*	*	*	*	*	*	*	*	*	*	*	*	*	*	*
Cl 3/PV218011.1/2024	*	*	*	*	*	*	*	*	*	*	*	*	*	R	*	*	*	*	*	*	*	*	*	*	*	*

Site I

residue number	23	24	25	26	27	28	29	30	31	282	283	284
Cl 1 consensus sequence	Y	L	E	E	S	C	S	T	I	P	C	W
Cl 1/EU857542.1/1982	*	*	*	*	*	*	*	*	*	*	*	*
Cl 1/OL794389.1/2006	*	*	*	*	*	*	*	*	*	*	*	*
Cl 2 consensus sequence	Y	L	E	E	S	C	S	T	I	P	C	W
Cl 2/EU857564.1/1986	*	*	*	*	*	*	*	*	*	*	*	*
Cl 2/OL794442.1/2016	*	*	*	*	*	*	*	*	*	*	*	*
Cl 3 consensus sequence	Y	L	E	E	S	C	S	T	I	P	C	W
Cl 3/JQ413393.1/2002	*	*	*	*	*	*	*	*	*	*	*	*
Cl 3/PV218011.1/2024	*	*	*	*	*	*	*	*	*	*	*	*

Site Ⅱ

residue number	233	234	235	236	237	238
Cl 1 consensus sequence	N	M	P	T	S	A
Cl 1/EU857542.1/1982	*	*	*	*	*	*
Cl 1/OL794389.1/2006	*	*	*	*	*	*
Cl 2 consensus sequence	N	M	P	T	S	A
Cl 2/EU857564.1/1986	*	*	*	*	*	*
Cl 2/OL794442.1/2016	*	*	*	*	*	*
Cl 3 consensus sequence	N	M	P	T	S	A
Cl 3/JQ413393.1/2002	*	*	*	*	*	*
Cl 3/PV218011.1/2024	*	*	*	*	*	*

Site Ⅲ

residue number	277	278	279	280	281
Cl 1 consensus sequence	G	V	I	D	T
Cl 1/EU857542.1/1982	*	*	*	*	*
Cl 1/OL794389.1/2006	*	*	*	*	*
Cl 2 consensus sequence	G	V	I	D	T
Cl 2/EU857564.1/1986	*	*	*	*	*
Cl 2/OL794442.1/2016	*	*	*	*	*
Cl 3 consensus sequence	G	V	I	D	T
Cl 3/JQ413393.1/2002	*	*	*	*	*
Cl 3/PV218011.1/2024	*	*	*	*	*

Site IV

residue number	395	396	397	398	399	400	401	402	403	404	405
Cl 1 consensus sequence	N	R	V	G	I	I	K	Q	L	N	K
Cl 1/EU857542.1/1982	*	*	*	*	*	*	*	*	*	*	*
Cl 1/OL794389.1/2006	*	*	*	*	*	*	*	*	*	*	*
Cl 2 consensus sequence	N	R	V	G	I	I	K	Q	L	N	K
Cl 2/EU857564.1/1986	*	*	*	*	*	*	*	*	*	S	*
Cl 2/OL794442.1/2016	*	*	*	*	*	*	*	*	*	*	*
Cl 3 consensus sequence	N	R	V	G	I	I	K	Q	L	N	K
Cl 3/JQ413393.1/2002	*	*	*	*	*	*	*	*	*	*	*
Cl 3/PV218011.1/2024	*	*	*	*	*	*	*	*	*	*	*

Site V

residue number	136	137	138	139	140	141	142	143	144	145	146	147	148	149	150	151	152	153	154	155	156
Cl 1 consensus sequence	A	I	K	N	A	L	K	K	T	N	E	A	V	S	T	L	G	N	G	V	R
Cl 1/EU857542.1/1982	*	*	*	*	*	*	*	*	*	*	*	*	*	*	*	*	*	*	*	*	*
Cl 1/OL794389.1/2006	*	*	*	*	*	*	*	*	*	*	*	*	*	*	*	*	*	*	*	*	*
Cl 2 consensus sequence	A	I	K	N	A	L	K	T	T	N	E	A	V	S	T	L	G	N	G	V	R
Cl 2/EU857564.1/1986	*	*	*	*	*	*	*	K	*	*	*	*	*	*	*	*	*	*	*	*	*
Cl 2/OL794442.1/2016	*	*	*	*	*	*	*	*	*	*	*	*	*	*	*	*	*	*	*	*	*
Cl 3 consensus sequence	A	I	K	N	A	L	K	K	T	N	E	A	V	S	T	L	G	N	G	V	R
Cl 3/JQ413393.1/2002	*	*	*	*	*	*	*	*	*	*	*	*	*	*	*	*	*	*	*	*	*
Cl 3/PV218011.1/2024	*	*	*	*	*	*	*	*	*	*	*	*	*	*	*	*	*	*	*	*	*

Cl : Cluster

□ : Non-consensus residues

Figure S3. Cluster-specific consensus sequences and representative-strain sequences across the core regions of neutralizing antibody-binding sites. Amino acids differing from the consensus sequence in each cluster are indicated with black boxes. Asterisks (*) indicate positions where the amino acid is identical to the consensus sequence.

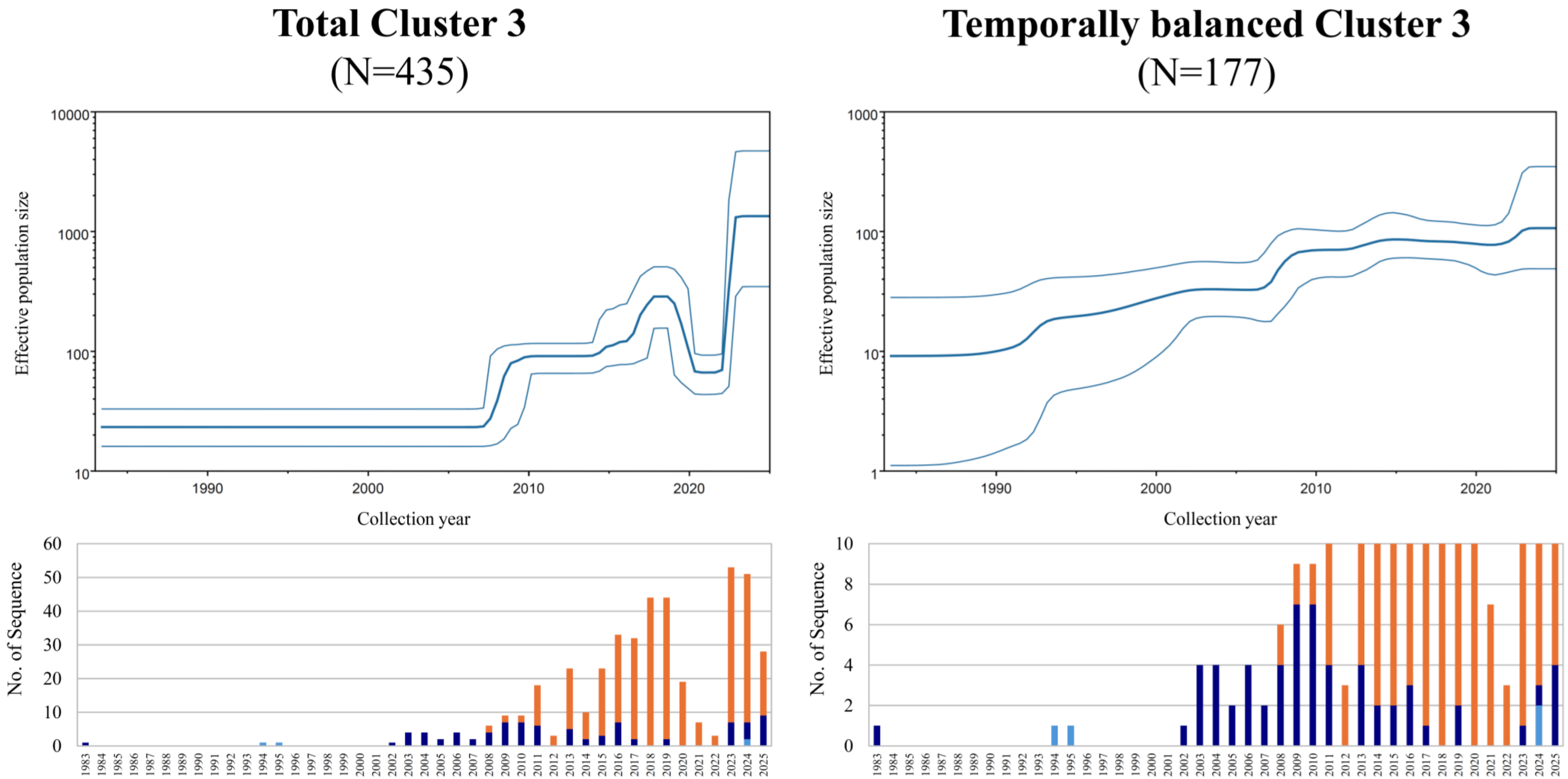

Figure S4. Comparison of Bayesian skyline trajectories and temporal lineage composition between the original and temporally balanced Cluster 3 datasets. The left panels show the original Cluster 3 dataset (*n* = 435), and the right panels show the temporally balanced Cluster 3 dataset (*n* = 177). In the upper panels, the thick line represents the posterior mean effective population size and the thin lines indicate the 95% highest posterior density (HPD) interval. In the lower panels, stacked bars show the annual numbers of sequences assigned by Nextclade to A2.2 (light blue), A2.2.1 (dark blue), and A2.2.2 (orange). For temporal balancing, all sequences were retained in years containing ≤10 sequences, whereas up to 10 phylogenetically representative sequences were retained using PARNAS in years containing >10 sequences. The y-axes are shown on different ranges between the original and temporally balanced datasets; therefore, the plots are intended primarily for comparison of temporal patterns rather than direct comparison of absolute effective population sizes or sequence counts.

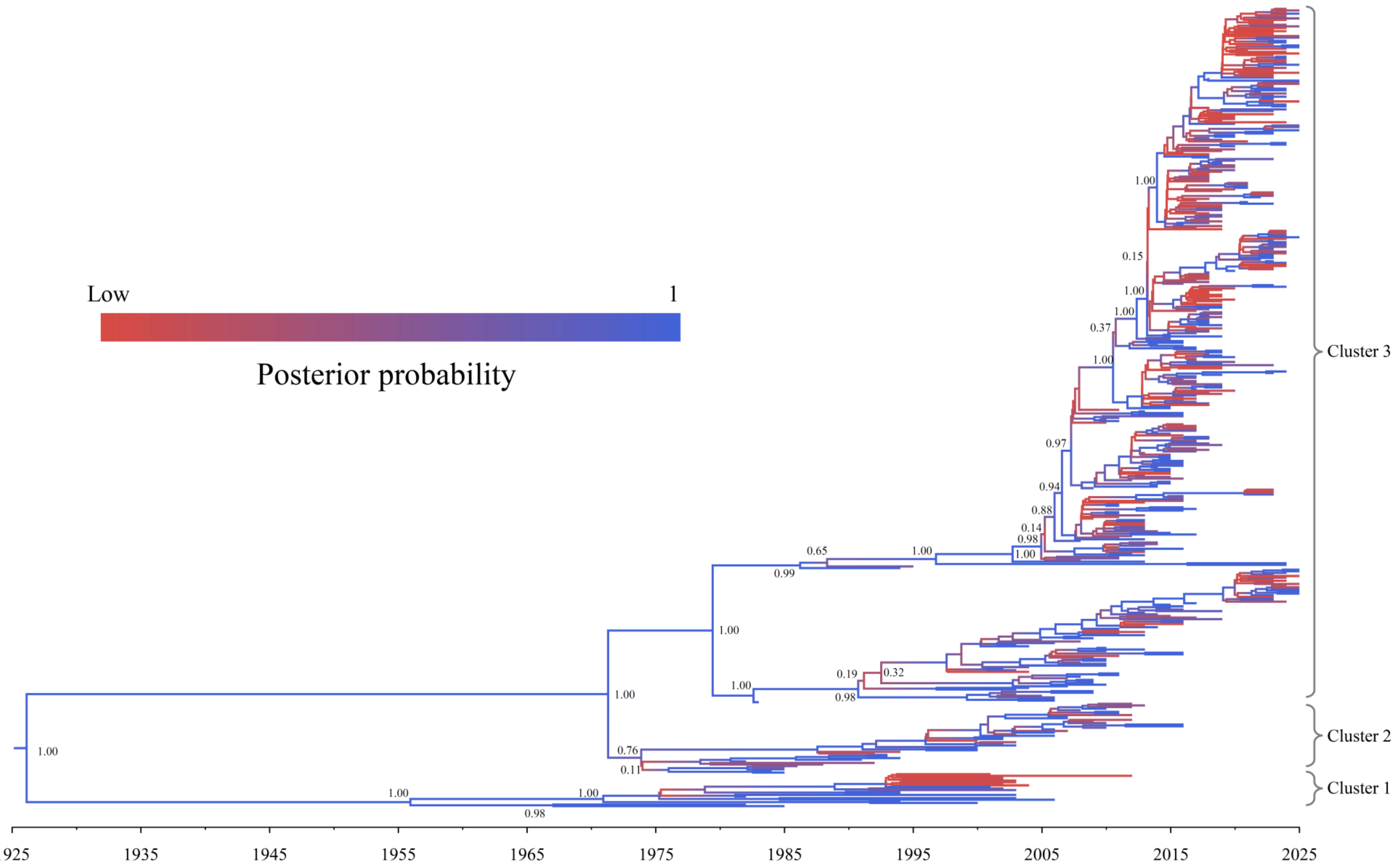

Figure S5. Time-scaled maximum clade credibility tree of HMPV-A F-gene sequences showing posterior probability support values. Posterior probability values are shown at internal nodes. Branch colors represent node posterior probabilities using an automatically scaled gradient from the minimum observed value (red) to 1.0 (blue). The three major phylogenetic groups identified in this study are indicated as Clusters 1–3. This tree corresponds to the MCC tree shown in Figure 1 and is provided to visualize posterior support for the inferred phylogenetic relationships.
